# KIF1A promotes neuroendocrine differentiation in prostate cancer by regulating the OGT-mediated O-GlcNAcylation

**DOI:** 10.1038/s41419-024-07142-2

**Published:** 2024-11-06

**Authors:** Qianqian Zhou, Muyi Yang, Jiawei Fu, Xinyu Sun, Jiajia Wang, Hanwen Zhang, Jing Hu, Bo Han

**Affiliations:** 1 The Key Laboratory of Experimental Teratology, Ministry of Education and Department of Pathology, School of Basic Medical Sciences, Cheeloo College of Medicine, Shandong University, Jinan, Shandong, 250000 P R China; 2 Department of Oncology-Pathology, Karolinska Institute, Stockholm, Sweden; 3 Jinan Central Hospital, Shandong University, Jinan, Shandong, 250000 P R China; 4 Department of Pathology, Shandong University Qilu Hospital, Jinan, Shandong, 250000 P R China

**Keywords:** Prostate cancer, Glycosylation

## Abstract

Neuroendocrine prostate cancer (NEPC) arises from prostate adenocarcinoma after endocrine treatment failure and implies lethality and limited therapeutic options. Deciphering the molecular mechanisms underlying transdifferentiation from adenocarcinoma to NEPC may provide valuable therapeutic strategies. We performed a pan-cancer differential mRNA abundance analysis and identified that Kinesin-like protein (KIF1A) was highly expressed in NEPC. KIF1A knockdown impaired neuroendocrine(NE) features, including NE marker gene expression, stemness, and epithelial–mesenchymal transition (EMT), whereas KIF1A overexpression promoted these processes. Targeting KIF1A inhibited the growth of NE differentiated prostate cancer (PCa) cells in vitro and in vivo. Mechanistically, KIF1A bound with O-linked N-acetylglucosamine transferase (OGT) and regulated its protein expression and activity. Nuclear accumulation of OGT induced by KIF1A overexpression promoted intranuclear O-GlcNAcylation of β-catenin and OCT4 in nucleus. More importantly, our data revealed that OGT was critical for KIF1A induced NE differentiation and aggressive tumor growth. An OGT inhibitor, OSMI-1, can significantly inhibited NE differentiated PCa cell proliferation in vitro and tumor growth in vivo. Our findings showed that KIF1A promotes NE differentiation to NEPC by regulating the OGT-mediated O-GlcNAcylation. Targeting O-GlcNAcylation may impede the development of NEPC for a group of PCa patients with elevated KIF1A expression.

## Background

Neuroendocrine prostate cancer (NEPC) is a highly aggressive form of prostate cancer (PCa), which is commonly characterized by typical neuroendocrine (NE) markers such as ENO2, SYP, and CHGA, but no or low levels of androgen receptor (AR) and AR-regulated genes [1, 2]. De novo NEPC occurs rarely, while nearly 20% metastatic castration-resistant prostate cancer (mCRPC) develop small-cell neuroendocrine pathologic features after potent endocrine therapy through a transdifferentiation mechanism [3]. NEPC tumors do not rely on AR signaling and are insensitive to endocrine therapy. Dissecting the molecular mechanisms of transdifferentiation from adenocarcinoma to NEPC may unveil targetable vulnerability and novel therapeutic strategies in NEPC.

There are multiple genetic alterations in NEPC, including mutations in *TP53*, *RB1*, and *PTEN* [4, 5], as well as amplification of *N-MYC*, *AURKA*, etc [6, 7]. *TP53* and *RB1* loss increases expression of NE markers, stem cell reprogramming factors, and epigenetic regulators, which leads to epigenetic reprogramming toward a stem cell-like state and ultimately facilitates NEPC formation [4, 5]. Lee et al. demonstrated that N-MYC drives NEPC initiated from human prostate epithelial cells and Dardenne et al. indicated that N-MYC drives the NEPC phenotype and EMT molecular program [8, 9]. Meanwhile, other drivers/regulators of NEPC also appear to be overexpressed, including MUC-1, BRN2, SOX2, ONECUT2, EZH2, SRRM4, SPINK1, etc [9–13]. MUC1-C facilitates NEPC progression by integrating activation of the MYC and NF-κB p65 pathways with inhibition of p53 to promote epithelial–mesenchymal transition (EMT), stemness and NE transdifferentiation [10]. Bishop et al. identified BRN2 as a major driver of NEPC, which promotes NE differentiation by synergistically regulating neural progenitor cells specific targets such as OCT4 and SOX2 [14, 15]. However, the mechanism underlying transdifferentiation of CRPC and the development to more-aggressive NEPC remains largely uncharacterized. Here, we integrated a selected panel of datasets and performed a comprehensive pan-cancer analysis to identify potential important genes of NE differentiation, and identified Kinesin-like protein (KIF1A) as a potential regulator to promote NE differentiation.

KIF1A is a major axonal transport motor protein, involved in the selection and regulation of dense core vesicle, lysosomal and synaptic vesicle trafficking [16, 17]. It has been reported that KIF1A is an important regulator of nervous system [18, 19]. The group of neurological disorders caused by mutations in the *KIF1A* gene in human are known as KIF1A-associated neurological disorders, with a diverse range of signs, manifesting as mental retardation, spasticity and epileptic seizures [20]. Of note, overexpression of KIF1A has been shown to be associated with a variety of malignancies. For example, elevated KIF1A is associated with poor survival prognosis and immune infiltration in ovarian cancer [21]. Intriguingly, we found that KIF1A was significantly upregulated in NE transdifferentiation of PCa. We established a substantial relationship between KIF1A and OGT mediated O-GlcNAcylation in transdifferentiation process. Moreover, KIF1A mediated aggressive growth and transdifferentiation could be blocked by O-GlcNAcylation inhibitor, suggesting that O-GlcNAcylation-directed therapy may benefit patients with NEPC.

## Methods

### Patients and tissue specimens

This study included two cases of PCa from Shandong University Qilu Hospital (Jinan, China) in 2021, which progressed from adenocarcinoma to NEPC after treatment with androgen deprivation therapy (ADT). The PCa biopsy specimens before and after treatment were used for immunohistochemistry. Moreover, 5 small cell lung cancer (SCLC) and 5 non-small cell lung cancer (NSCLC) cases were also included in our study. This study was approved by Shandong University Medical Research Ethics Committee (Document No. ECSBMSSDU2021-1-61) and informed consent was obtained from each patient.

### Cell lines and regents

Human PCa cell lines (DU145, LNCaP, C4-2B, 22RV1 and NCI-H660) and HEK293T were obtained from the American Type Culture Collection (ATCC, Virginia, USA) and cultured following the manufacturer’s recommendations. LNCaP-AI cells were established by culturing LNCaP cells in phenol red-free RPMI1640 medium (Thermo Fisher Scientific, USA) containing 10% charcoal-stripped FBS (Gibco, USA) for over 12 months. Routine contamination with mycoplasma was detected using the PlasmoTest Mycoplasma Test Kit (InvivoGen, USA). Authentication of the cells was performed by short tandem repeat analysis. Cells were treated with the protein synthesis inhibitor cycloheximide (Sigma-Aldrich, USA), proteasome inhibitor MG132 (MedChemExpress, USA), the AR pathway inhibitor enzalutamide (MedChemExpress, USA) or docetaxel (MedChemExpress, USA).

### Plasmids and cell transfection

KIF1A (Gene ID: 547; vector: PcDNA3.1), OGT (Gene ID: 8473; vector: PcDNA3.1) cDNA expression vectors were purchased from Biosune Biotech. Lipofectamine 3000 (Invitrogen, USA) was used following the manufacturer’s instructions. The effect of transfection efficiency was confirmed by Western blot. Cell lines that constitutively express control shRNA, shKIF1A were selected by 2 mg/mL puromycin in the culture medium for 2 weeks. Human Lenti-OGT and its control were obtained from Genecopoeia. Blasticidin was used to select LNCaP-AI cells for stable overexpression of OGT. siRNA and shRNA sequences were shown in Supplementary Table 1. The effect of transfection efficiency was confirmed by quantitative real‑time PCR and Western blot. Information of the primers and antibodies used in the tests were shown in Supplementary Table 2–3.

### Immunohistochemistry

Immunohistochemistry was carried out as described previously [22, 23]. The tissue slides were incubated with the indicated primary antibodies overnight at 4 °C. Primary antibodies were listed in Supplementary Table 3. More detailed information of the immunohistochemistry procedure was included in Supplementary Materials and Methods.

### Xenograft studies in nude mice

4-6-week-old male nude mice were purchased from Weitonglihua Biotechnology (Beijing, China). 1 × 10^7^ LNCaP-AI cells with shSCR/shKIF1A/shKIF1A+OGT treatment were suspended in 100 μl of PBS with 50% matrigel and injected subcutaneously into the mice. A xenograft model was used to assess the effect of OSMI-1 on tumor growth in vivo. LNCaP-AI cells were subcutaneously injected into nude mice and the nude mice were randomly divided into control (DMSO), OSMI-1 (1 mg/kg, intravenously) after 7 days (n = 6/group). Each group was intravenously administered every other day for 4 weeks. Tumor volume (0.5×length×width^2^) was measured twice a week. The experimental protocols were performed following the Ethical Animal Care and Use Committee of Shandong University (Document No. ECSBMSSDU2021-2-126).

### RNA sequencing and bioinformatics analysis

Microarray-based human gene expression profiling (Kang Cheng, Shanghai, China) was used to compare the mRNA expression profiles of LNCaP and LNCaP-AI cells. The RNA-sequence and microarray data in this study have been deposited in Gene Expression Omnibus (GEO) (http://www.ncbi.nlm.nih.gov/geo) with the accession number GSE266283. The expressed genes were analyzed for enrichment of biological themes using Gene Set Enrichment Analysis (GSEA; http://software.broadinstitute.org/gsea/index. jsp). Datasets of GSE104786, GSE32967, GSE59986, GSE90891, GSE118207, GS40275, GSE11969 and GSE30219 were downloaded from the GEO database (http://www.ncbi.nlm.nih.gov/geo). KIF1A expression in NSCLC and SCLC cell lines was downloaded from Cancer Cell Line Encyclopedia(https://sites.broadinstitute.org/ccle/). Beltran 2016 and SU2C 2019 datasets were downloaded from cBioPortal for Cancer Genomics(https://www.cbioportal.org/). The heatmap was visualized using algorithms at Bioinformatics (https://www.bioinformatics.com.cn/).

### Statistical analysis

All results were expressed as the mean ± SD. Statistical analysis was carried out using GraphPad Prism 7 or the SPSS 20.0 software. One-way ANOVA or two-tailed unpaired *t* test was used to calculate statistical significance between groups. Correlation between two expression groups was measured by Pearson’s r. *p* values were considered to be significant as follows: *p <0.05; **p <0.01; ***p <0.001 and ****p <0.0001.

## Results

### KIF1A overexpression is associated with NE phenotype

**Fig. 1:**
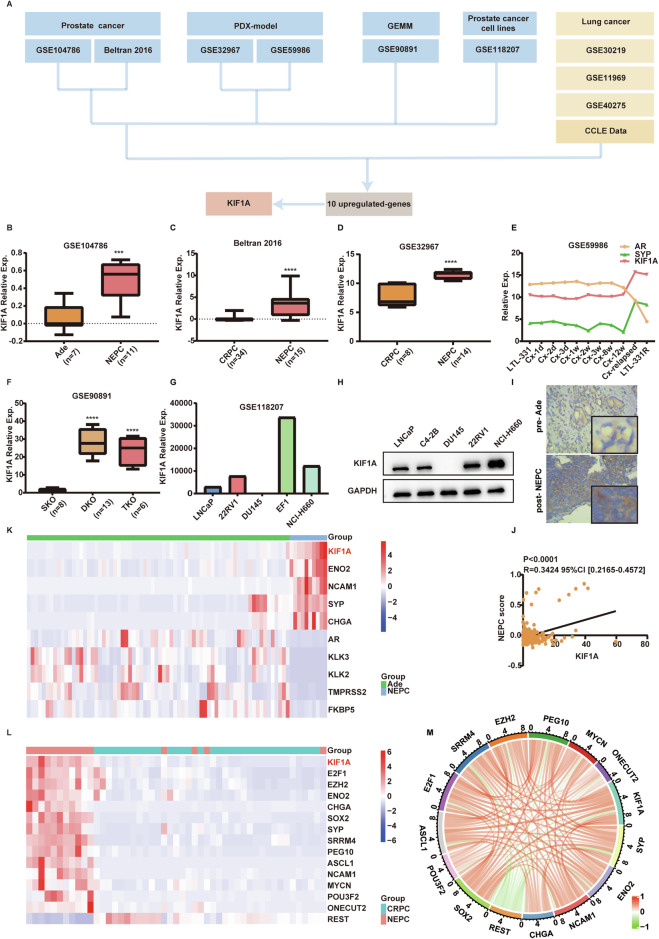
KIF1A overexpression is associated with NE phenotype. **A** A summary of dataset integration and the pipeline for putative driver gene selection. **B**, **C** Expression of *KIF1A* in NEPC patient samples compared with CRPC or Ade in GSE104786, Beltran 2016 dataset. **D** Expression of *KIF1A* in NEPC PDX models compared with CRPC. **E** Expressions of *KIF1A*, *AR*, and *SYP* during the transformation from Ade (LTL331) to NEPC (LTL331R) after castration in GSE59986 dataset. **F** Expressions of *KIF1A* in different GEMMs in GSE90891 dataset. **G** The expressions of KIF1A across different PCa cell lines in GSE118207 dataset. **H** KIF1A protein levels in LNCaP, C4-2B, DU145, 22RV1 or NCI-H660 cells. **I** Representative images showing immunohistochemistry staining for KIF1A in Ade and NEPC cases. **J** The Pearson’s *r* correlation coefficient between *KIF1A* and NEPC score in SU2C 2019 cohort. NEPC score was calculated as described previously [24]. **K** Heatmap showing expression of KIF1A, AR signal pathway and NE associated genes in GSE126078 dataset. **L** Heatmap showing expression of KIF1A and NE associated genes in Beltran 2016 cohort. **M** Circos plot displaying the interconnectivity among NE associated genes and KIF1A. The thickness and color of the strip indicated the correlation coefficient in Beltran 2016 cohort. All results were presented as the mean ± SD. ***p <0.001, ****p <0.0001, based on Student’s t test. PDX patient-derived xenograft, GEMM genetically engineered mouse model, Ade adenocarcinoma.

To identify potential important genes related to NEPC, we performed differential mRNA abundance analysis in four different NEPC datasets including patient samples, patient-derived xenograft (PDX) models, genetically engineered mouse model (GEMM) and cell lines. Details about the bioinformatic analysis were reported in the Supplementary Fig. 1. Finally, fifteen genes were up-regulated in NEPC samples compared to controls across these four datasets (Supplementary Fig. 1). Considering the shared genomic and epigenetic alteration features between NEPC and SCLC, we next performed validation of these 15 candidate genes in three independent datasets containing SCLC patient samples as well as the Cancer Cell Line Encyclopedia (CCLE) dataset. Of these 15 candidates, ten genes were up-regulated (Fold change>1.5 & *p* <0.05) in SCLC relative to NSCLC (Fig. 1A, Supplementary Fig. 1). In order to identify the most plausible molecules, we calculated the effect sizes (Cohen’s *d*) of these ten molecules individually in Beltran 2016 cohort, thus filtering out the molecules with the highest differences between NEPC and CRPC. Among them, *KIF1A*, which had the largest Cohen’s *d* value in landmark NEPC dataset [24], was selected for further analyses (Fig. 1A, Supplementary Table 4). As shown in Fig. 1B, KIF1A expression was higher in NEPC patient samples than those of localized prostatic adenocarcinoma. Meanwhile, compared to CRPC, the expression of KIF1A was increased in NEPC patient samples and PDX models (Fig. 1C, D). Particularly, KIF1A was up-regulated during the construction of LTL331R PDX model which mimicked the progression of PCa from adenocarcinoma to NEPC through castration (Fig. 1E). Transcriptome data showed that KIF1A was strongly up-regulated in DKO and TKO GEMMs, which developed aggressive PCa with increased lineage plasticity and NE features than *PTEN* knockout (SKO) GEMMs (Fig. 1F). The expression of KIF1A in NEPC cell lines EF1 and NCI-H660 was higher than that in other PCa cell lines (Fig. 1G). In the before mentioned four SCLC datasets, *KIF1A* expression was up-regulated in SCLC compared to NSCLC (Supplementary Fig. 2A–D). We detected KIF1A expression in PCa cell lines and found that KIF1A expression was higher in NEPC cells NCI-H660 compared to other PCa cells LNCaP, DU145, C4-2B and 22RV1 (Fig. 1H). Importantly, we analyzed KIF1A expression by immunohistochemistry in two PCa cases, where the pre-endocrine treatment tissue was adenocarcinoma, while the post-treatment tissue displayed NEPC. Immunohistochemistry showed that KIF1A expression was elevated in post-treatment NEPC tissue compared to pre-treatment adenocarcinoma tissue (Fig. 1I). These data suggested that KIF1A expression was significantly upregulated in NEPC. In addition, bioinformatic analysis showed that *KIF1A* was positively related with NEPC score (Fig. 1J, SU2C 2019) and NE markers (Fig. 1K, Labrecque 2019; Fig. 1L, Beltran 2016). *KIF1A* also showed positive correlation with genes promoting NE feature (*SOX2*, *SRRM4*, etc.) and negative correlation with NE feature repressor *REST* (Fig. 1L, M, Supplementary Fig. 2E–S). Collectively, these data suggested that *KIF1A* is up-regulated in NEPC and associated with NE features.

### KIF1A overexpression induces NEPC phenotype

**Fig. 2:**
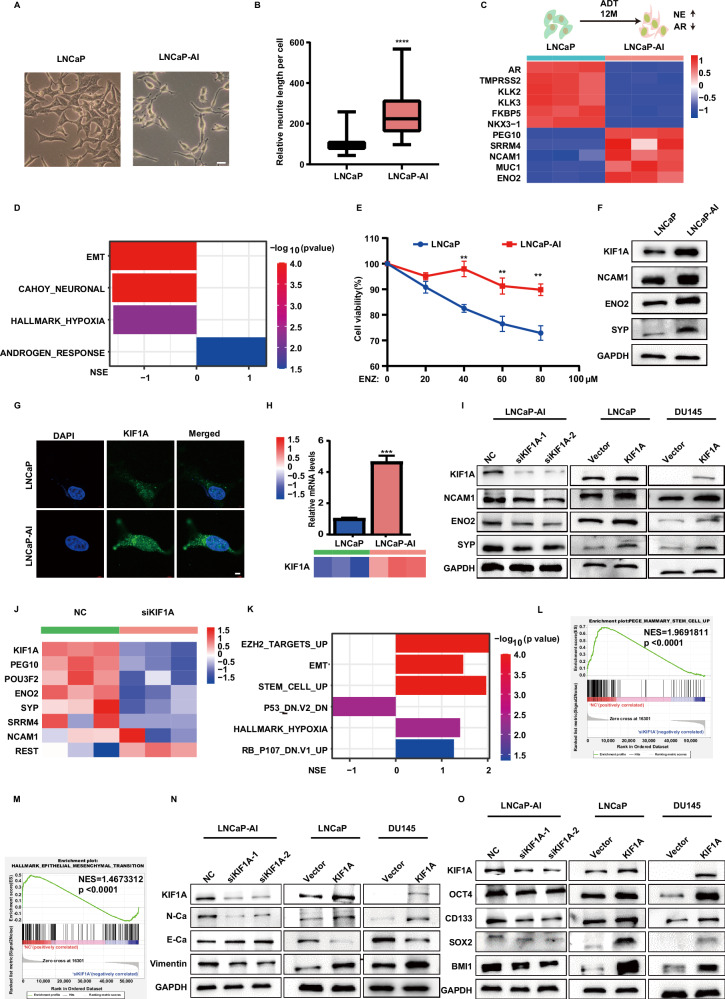
KIF1A overexpression induces NEPC phenotype. **A** The morphology of cells was imaged by Zeiss light microscope, Scale bar = 20 μm. **B** Quantitative result of protrusions length in LNCaP or LNCaP-AI cell. **C** Heatmap of AR signaling and NE associated genes in LNCaP cells or LNCaP-AI cells. **D** Gene sets significantly enriched in LNCaP cells compared to LNCaP-AI cells. NES and *p* value were reported. **E** Cell viability measured in the indicated cell lines by CCK-8 assay. LNCaP and LNCaP-AI cells were treated with titrated doses of ENZ for 3 days. **F** NE markers and KIF1A protein levels in LNCaP or LNCaP-AI cell. **G** Representative images of immunofluorescence of KIF1A in LNCaP or LNCaP-AI cells. Cells were imaged by confocal microscopy. Scar bar = 5 μm. **H** The mRNA levels of KIF1A in LNCaP/LNCaP-AI cells. The mRNA was first measured by RNA-seq (lower panel) and then validated through qPCR (upper panel). **I** Western blot of NE markers (NCAM1, ENO2, SYP) and KIF1A in indicated PCa cells with KIF1A overexpression or knockdown. **J** Heatmap showing NE associated genes expression in indicated LNCaP-AI cells. siKIF1A-2 were utilized as siKIF1A. **K** Gene sets significantly enriched in high- KIF1A group (NC) compared to siKIF1A in LNCaP-AI cells. NES and *p*value were reported. **L** GSEA of the EMT hallmark genes in NC and siKIF1A LNCaP-AI cells. **M** GSEA of the stem cell up-regulated genes in NC and siKIF1A LNCaP-AI cells. **N** Western blot of KIF1A and EMT markers in indicated PCa cells with KIF1A overexpression or knockdown. **O** Western blot of KIF1A and stemness related genes in indicated PCa cells with KIF1A overexpression or knockdown. All results were presented as the mean ± SD. ****p <0.0001, based on Student’s t test. ADT androgen deprivation therapy, ENZ enzalutamide, NSE normalized enrichment score.

To characterize the role of KIF1A in NEPC phenotype, we established LNCaP-AI cell line by culturing androgen-sensitive LNCaP cells in androgen deprivation conditions for one year as previously described [13, 25]. We observed a morphological change from an epithelial phenotype to a NE-like phenotype, characterized by an increase of the nuclear to cytoplasmic ratio and an approximately fourfold increase in the length of neuron-like dendritic protrusions (Fig. 2A, B). Compared to LNCaP cells, LNCaP-AI cells demonstrated down-regulated AR signaling, increased NE marker expression and NEPC genomic feature, including positive enrichment of neuronal, EMT and hypoxia genes (Fig. 2C, D, Supplementary Fig. 3A–E). LNCaP-AI cells also showed enhanced proliferation, migration, invasion capabilities (Supplementary Fig. 3F–H). More importantly, LNCaP-AI cells were less sensitive to enzalutamide and docetaxel compared to LNCaP cells (Fig. 2E, Supplementary Fig. 3I). Of note, both protein and mRNA levels of KIF1A were significantly elevated in LNCaP-AI cells compared to LNCaP cells (Fig. 2F–H).

We then applied small interfering RNA to deplete KIF1A mRNA in LNCaP-AI cells and overexpressed KIF1A expression in LNCaP and DU145 cells. KIF1A knockdown decreased expression of NE marker (NCAM1, ENO2, and SYP) in LNCaP-AI cells, while KIF1A overexpression increased those protein expression in LNCaP and DU145 cells (Fig. 2I). Transcriptome sequencing was used to compare the mRNA expression profiles in control (NC) and KIF1A knockdown (siKIF1A) LNCaP-AI cells. Compared with control, KIF1A knockdown reduced mRNA levels of NE associated genes (ENO2, SYP, POU3F2, SRRM4, etc.), while it increased the mRNA level of REST (Fig. 2J). GSEA showed that *P53* and *RB* down-regulated genes, EZH2 target genes and hypoxia related genes, which were associated with NEPC, were enriched in the control cells (Fig. 2K, Supplementary Fig. 4A–D). Of note, GSEA revealed that EMT related genes (*p* <0.0001) and stem cell up-regulated genes (*p* <0.0001) were significantly enriched in the control cells (Fig. 2L-M). Meanwhile, knockdown of KIF1A increased the expression of E-cadherin (epithelial marker), but decreased the expression of Vimentin and N-cadherin (mesenchymal marker) in LNCaP-AI cells (Fig. 2N). Similarly, a significant decrease of E-Cadherin and increase of Vimentin and N-Cadherin were observed in KIF1A overexpressed LNCaP and DU145 cell compared to control, highlighting an important role of KIF1A in EMT (Fig. 2N). Moreover, knockdown of KIF1A in LNCaP-AI cells resulted in downregulation of stemness related molecules, while overexpression of KIF1A in LNCaP and DU145 cells induced expression of those molecules (Fig. 2O). Collectively, these data suggested that KIF1A promotes the stemness and EMT, which in turn contributed to NEPC phenotype.

### KIF1A is required for aggressive growth of NE transdifferentiated PCa cell in vivo and in vitro

**Fig. 3:**
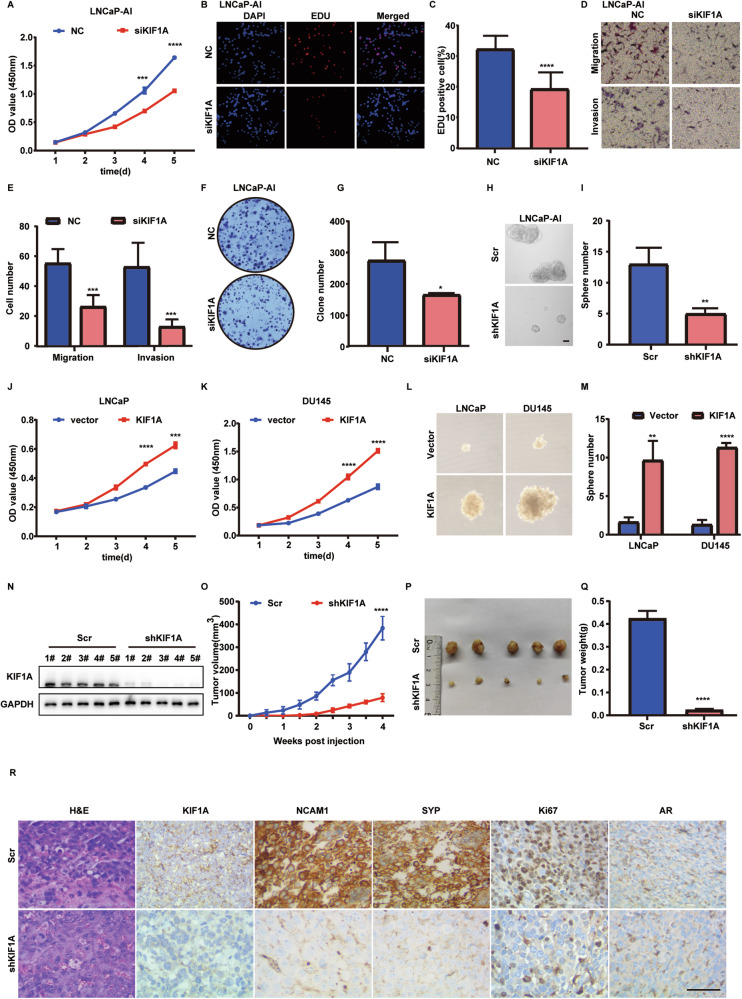
KIF1A is required for aggressive growth of NE transdifferentiated PCa cell in vitro and in vivo. **A** Cell viability assessed by CCK-8 assay after transfection at different time points in LNCaP-AI cells. NC/siKIF1A: KIF1A was knockdown in LNCaP-AI cells by transfection of siRNA targeting KIF1A (siKIF1A) or a negative control (NC). **B**, **C** Representative images and quantitative results of EDU assay in indicated LNCaP-AI cells from three independent experiments. **D**, **E** Representative images and quantitative results of transwell migration and matrigel invasion assays in LNCaP-AI cells with KIF1A ablation from three independent experiments. **F** Colony formation assay of indicated LNCaP-AI cells. **G** Quantitative analysis of colony number from three independent experiments. **H** Sphere formation assay of LNCaP-AI cells. **I** Quantitative results of sphere formation assays in LNCaP-AI cells from three independent experiments. Indicated cells were dissociated to single cells under suspension culture conditions in the presence of 20 ng/ml EGF, 10 ng/ml bFGF, and 2% B27 supplement. Spheroids with diameter >75 μm were counted. Scale bar = 40 μm. Scr/shKIF1A: LNCaP-AI cells were transfected with shRNA targeting KIF1A (shKIF1A) or a negative control (Scr). **J**, **K** Cell viability assessed by CCK-8 assay after transfection at different time points in indicated PCa cells. **L** Sphere formation assay of LNCaP and DU145 cells. **M** Quantitative results of sphere formation assays in LNCaP and DU145 cells from three independent experiments. **N**, **O** Effect of KIF1A on tumorigenesis in vivo evaluated with xenografts model. LNCaP-AI cells with stable expression of Scr/shKIF1A subcutaneously injected into nude mice. Measurement of stable transfection efficiency (**N**), The growth curve (**O**), Representative images of xenograft tumors (**P**), Tumor weight (**Q**). **R** Representative images showing H&E staining and immunostaining for KIF1A, Ki67, SYP, NCAM1 and AR in LNCaP-AI xenograft tumors. Scale bar = 100 μM. All results were presented as the mean ± SD. of three independent experiments. *p <0.05, **p <0.01, ***p <0.001, ****p <0.0001, based on Student’s t test.

We subsequently investigated whether KIF1A expression affects the aggressiveness of LNCaP-AI cells. Knockdown of KIF1A significantly inhibited cell proliferation assessed by CCK-8 assay (Fig. 3A) and EDU assays (Fig. 3B, C). Inhibition of KIF1A also led to the decrease of cell migration, invasion, colony formation and sphere formation ability in LNCaP-AI cells (Fig. 3D–I). Overexpression of KIF1A in LNCaP and DU145 cells resulted in accelerated cell proliferation, migration, invasion and sphere formation compared to control (Fig. 3J–M, Supplementary Fig. 5A–D). Furthermore, KIF1A depletion in LNCaP-AI cells xenografts in nude mice inhibited growth of xenograft tumors (Fig. 3N–R). The mean tumor volume in the LNCaP-AI xenografts with KIF1A knockdown was 79.15 ± 16.83 mm^3^, while the control group was 383.5 ± 51.69 mm^3^ (*p* <0.0001) (Fig. 3O, P). The mean tumor weight in the LNCaP-AI xenografts with KIF1A knockdown was 0.0226 ± 0.0056 g, which was significantly lower than that in the control group with the mean tumor weight of 0.4234 ± 0.03369 g (*p* <0.0001) (Fig. 3Q). H&E staining showed that the xenograft tumors derived from LNCaP-AI cells displayed morphological features resembling small cell neuroendocrine carcinoma, including hyperchromatic nucleus and decreased cytoplasm of tumor cells and more necrosis than xenograft with KIF1A knockdown (Fig. 3R, Supplementary Fig. 5E). Meanwhile, immunohistochemistry for KIF1A, AR, Ki67, NCAM1 and SYP was performed in LNCaP-AI cells xenograft tumors (Fig. 3R). Xenografts from both groups exhibited similar low intensity of AR staining, while NE markers and the Ki67 index were decreased in the shKIF1A group (Fig. 3R). In summary, these results revealed that KIF1A acts as a promoter of aggressive cancer progression in NE transdifferentiated PCa cells.

### KIF1A binds to OGT and stabilizes OGT protein

**Fig. 4:**
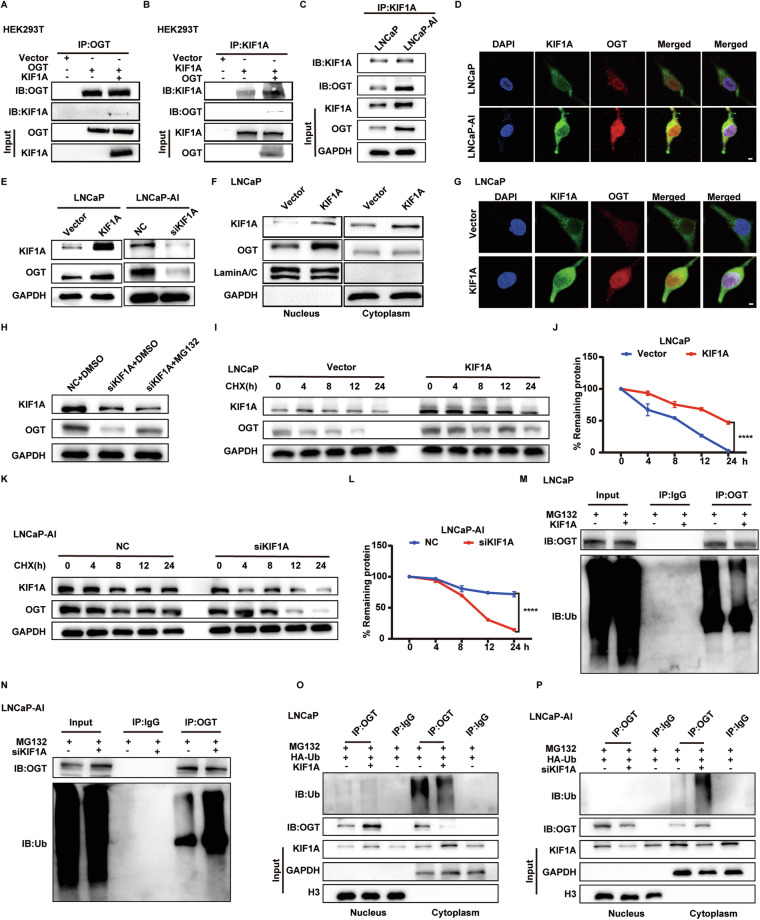
KIF1A binds to OGT and stabilizes OGT protein. **A**–**C** Co-IP assays of KIF1A and OGT in HEK293T and LNCaP-AI cells. **D** Representative images of immunofluorescence detection of KIF1A (green) and OGT (red) in LNCaP and LNCaP-AI cells. Cells were imaged by confocal microscopy. Scale bar = 5 μm. **E** Western blot of OGT and KIF1A in indicated PCa cells with overexpression/knockdown of KIF1A. **F** Nuclear/cytoplasmic expression of OGT and KIF1A in LNCaP cells with KIF1A overexpression. **G** Representative images of immunofluorescence detection of KIF1A (green) and OGT (red) in LNCaP cells with KIF1A overexpression. Cells were imaged by confocal microscopy. Scale bar = 5 μm. **H** Western blot of KIF1A and OGT in LNCaP-AI cells with knockdown of KIF1A. After 48 h of transfection, LNCaP-AI cells were treated with 20 μM MG132 or an equivalent dose of DMSO for 24 h. **I**–**L** Protein half-life assays in indicated PCa cells with knockdown/ overexpression of KIF1A. After 48 h of transfection, cells were treated with 10 μg/mL CHX and collected at 0, 4, 8, 12 and 24 h. The protein levels of OGT were determined by Western blot and the densitometry of OGT was normalized by GAPDH of three independent experiments. **M**, **N** IP and Western blot of OGT ubiquitination in indicated PCa cells with knockdown/ overexpression of KIF1A. **O**, **P** IP and Western blot of OGT ubiquitination extracted from nucleus/cytoplasm in indicated PCa cells with knockdown/ overexpression of KIF1A. LNCaP/LNCaP-AI cells were treated with 20 μM MG132 for 24 h. ***p <0.001, based on Student’s t test.

The AR signaling pathway plays a crucial role in PCa, and many AR repressive genes (e.g. SPINK1) promote NE transdifferentiation under ADT [13]. However, KIF1A expression was not regulated by AR signaling and vice versa (Supplementary Fig. 6A–D). To explore the molecular mechanism, we performed Immunoprecipitation (IP) and mass spectrometry analysis to screen for KIF1A-binding proteins in LNCaP-AI cells. A total of 1333 proteins bound to KIF1A were identified (Supplementary Table 5), and 57 proteins were determined to be neurologically related by querying protein functions using the Uniprot database (https://www.uniprot.org/). Four of those molecules were associated with stemness, including PRDX1, ANP32E, KDM1A and OGT, while only KDM1A and OGT had previously been shown to regulate EMT [26]. Immunofluorescence and coimmunoprecipitation (Co-IP) assays confirmed that KIF1A bound to OGT in HEK293T, LNCaP and LNCaP-AI cells (Fig. 4A–D). Of note, the interaction between KIF1A and OGT was more abundant in LNCaP-AI cells compared to LNCaP cells. Moreover, Western blot results and immunofluorescence assays showed an increase of OGT expression in LNCaP-AI cells (Fig. 4C, D). Overexpression of KIF1A induced upregulation of OGT in LNCaP cells, while knockdown of KIF1A resulted in downregulation of OGT in LNCaP-AI cells (Fig. 4E). We further interrogated the subcellular location of OGT protein induced by KIF1A. As demonstrated in Fig. 4F, G, KIF1A overexpression induced upregulation of OGT in the nucleus, while no changes were observed in the cytoplasm (Fig. 4F, G). Knockdown of KIF1A mainly resulted in downregulation of intranuclear OGT in LNCaP-AI cells (Supplementary Fig. 7C, D). However, quantitative real‑time PCR showed that KIF1A did not alter mRNA expression level of OGT (Supplementary Fig. 7E, F), which suggested a plausible post-translational modification. The proteasome inhibitor MG132 blocked the regulation of KIF1A for OGT, suggesting that KIF1A modulated OGT protein in proteasome degradation process (Fig. 4H). Next, we found that overexpression of KIF1A significantly prolonged the half-life of OGT proteins in LNCaP cells, while down-regulated KIF1A decreased the stability of OGT protein (Fig. 4I–L). Furthermore, the ubiquitination of OGT was found to be reduced in LNCaP cells with KIF1A overexpression, while knockdown of KIF1A resulted in an increase of OGT ubiquitination (Fig. 4M, N). We also found that the ubiquitination of OGT occurred mainly in the cytoplasm, where KIF1A affected binding of ubiquitin to OGT (Fig. 4O, P). These data indicated that KIF1A enhances OGT protein stabilization by evading degradation via the ubiquitin-proteasome pathway.

### KIF1A regulates activity of OGT to promote intranuclear O-GlcNAcylation of OCT4 and β-catenin

**Fig. 5:**
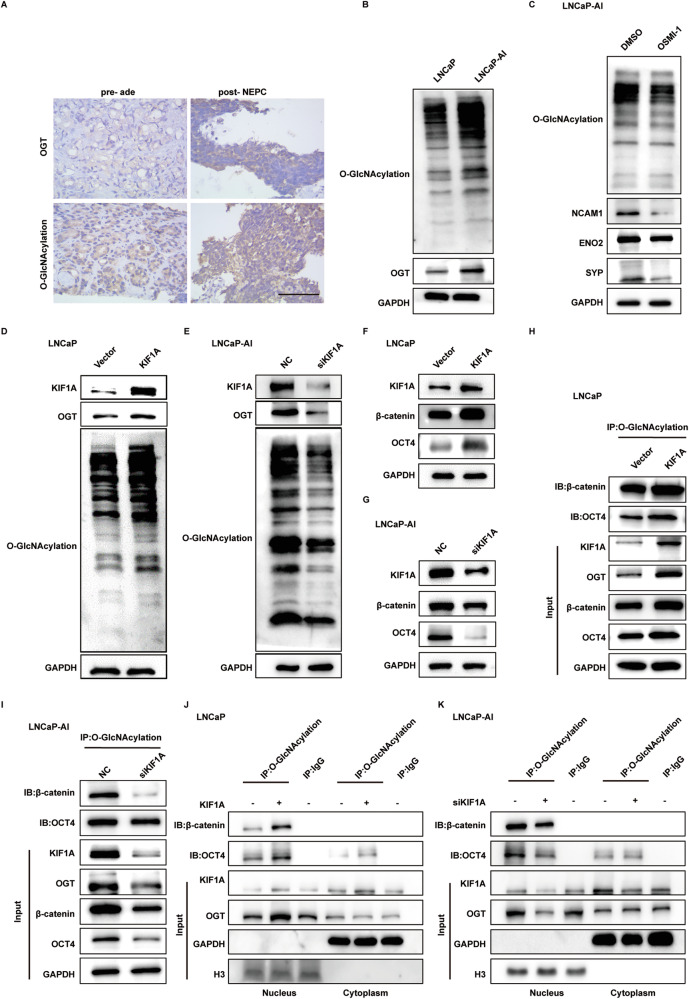
KIF1A regulates activity of OGT to promote intranuclear O-GlcNAcylation of OCT4 and β-catenin. **A** Representative images showing immunohistochemistry staining for O-GlcNAcylation and OGT in ade and NEPC. Scar bar = 100 μm. **B** Western blot of O-GlcNAcylation and OGT in LNCaP/LNCaP-AI cells. **C** O-GlcNAcylation and NE marker expression in LNCaP-AI cells measured by Western blot assays. LNCaP-AI cells were treated with 20 μM OSMI-1 or DMSO for 72 h. **D**, **E** Overall protein O-GlcNAcylation level in LNCaP/LNCaP-AI cells with KIF1A perturbation assessed by western blot assay. **F**, **G** Immunobloting analysis of indicated PCa cells showing changed in β-catenin and OCT4 protein level. **H**, **I** Detection of O-GlcNAcylated β-catenin and OCT4 protein in indicated PCa cells with overexpression or knockdown of KIF1A. **J**, **K** Subcellular detection of O-GlcNAcylated β-catenin and OCT4 in indicated PCa cells with overexpression/knockdown of KIF1A. ade, adenocarcinoma.

To the best of knowledge, OGT is the only unequivocally O-glycosyltransferase [27, 28]. We further investigated OGT expression and O-GlcNAcylation level in NEPC. OGT mRNA levels were not statistically different in NE-related groups versus control in NEPC datasets (Supplementary Fig. 8). However, immunohistochemistry of pre- and post-treatment specimens from two NEPC patients showed that both O-GlcNAcylation levels and OGT expression were elevated in post-treatment NEPC tissue compared to pre-treatment adenocarcinoma tissue (Fig. 5A). Similar results were observed in SCLC cases when compared to NSCLC patients (Supplementary Fig. 9). Meanwhile, LNCaP-AI cell also exhibited higher whole protein O-GlcNAcylation levels and OGT expression in comparison with LNCaP cells (Fig. 5B). Furthermore, treatment of OSMI-1, an inhibitor of OGT O-glycosyltransferase activity, down-regulated protein expression of NE marker in LNCaP-AI cells (Fig. 5C), highlighting the plausible role of OGT and O-GlcNAcylation in NE transdifferentiation.

Overexpressed KIF1A in LNCaP cells not only elevated expression of OGT but also the overall O-GlcNAcylation level, while knockdown of KIF1A decreased that in LNCaP-AI cells (Fig. 5D, E). Perturbation of KIF1A expression also affected β-catenin and OCT4 expression and O-GlcNAcylation, both of which were modified by OGT in NE transdifferentiation process (Fig. 5F–I). Western blot results showed increased β-catenin and OCT4 expression as well as O-GlcNAcylation in KIF1A overexpressed LNCaP cells, while attenuated expression and O-GlcNAcylation in cells with KIF1A ablation (Fig. 5F–I). Given that KIF1A induced OGT nuclear accumulation, we sought to evaluate whether KIF1A would elicit O-GlcNAcylation of protein in nucleus. Overexpression of KIF1A in LNCaP cells resulted in an increase in the intranuclear O-GlcNAcylation levels of OCT4 and β-catenin (Fig. 5J), while knockdown of KIF1A decreased intranuclear O-GlcNAcylation levels of OCT4 and β-catenin in LNCaP-AI cells (Fig. 5K). These results suggested that KIF1A regulates activity of OGT and promotes intranuclear O-GlcNAcylation of OCT4 and β-catenin.

### OGT is essential for KIF1A induced NE phenotype and aggressive growth

**Fig. 6:**
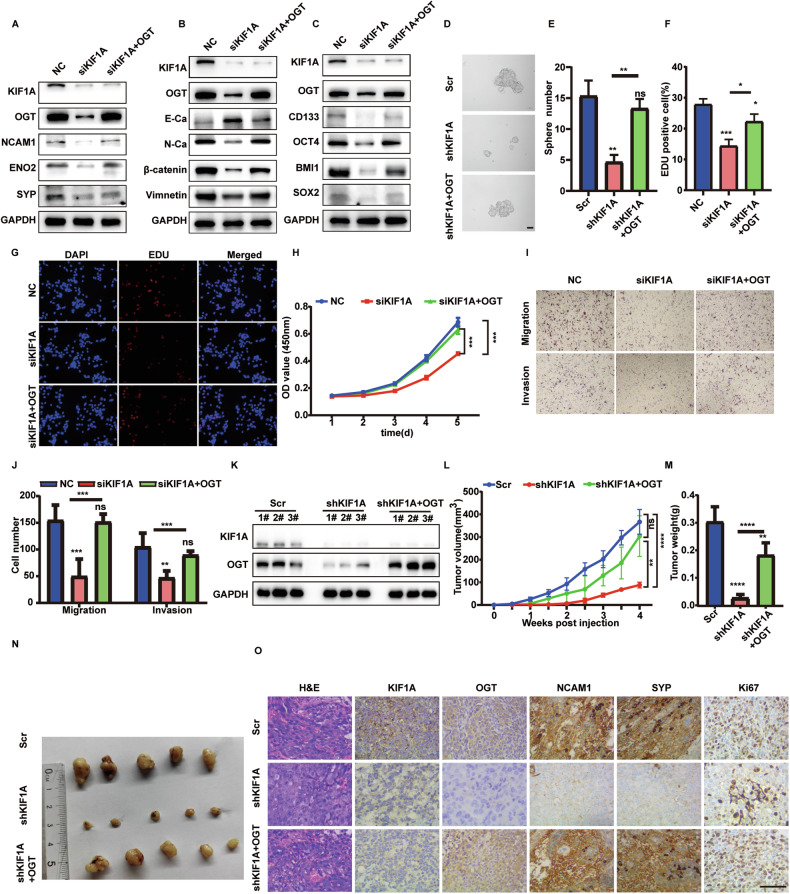
OGT is essential for KIF1A induced NE transdifferentiation and aggressive growth. **A**–**C** Western blot of indicated NE, EMT and stemness markers in LNCaP-AI cells upon KIF1A knockdown with or without OGT ectopic expression. **D**, **E** Representative image and quantification of sphere formation assay of indicated LNCaP-AI cells. LNCaP-AI cells stably transfected KIF1A shRNA with or without OGT ectopic expression were performed to sphere formation assays. Spheroids with diameter >75 μm were counted. Scale bars = 40 μm. **F**, **G** Representative images and quantification of EDU assay in indicated LNCaP-AI cells. **H** Cell viability assessed by CCK-8 assay at different time points in indicated LNCaP-AI cells. **I**, **J** Representative images and quantification of transwell migration and matrigel invasion assays in indicated LNCaP-AI cells. **K**–**M** Effect of KIF1A-OGT axis on tumorigenesis in vivo evaluated with xenografts model. LNCaP-AI cells stably transfected Scr/ shKIF1A /shKIF1A + OGT subcutaneously injected into nude mice. Measurement of stable transfection efficiency (**K**), The growth curve (**L**), Tumor weight (**M**), Representative image of xenograft tumors (**N**). **O** Representative images showing H&E staining and immunostaining for KIF1A, OGT, Ki67, SYP, NCAM1 in LNCaP-AI xenograft tumors as described in **K**–**M**. Scar bar = 100 μm. All results were presented as the mean ± SD. of three independent experiments. *p <0.05, **p <0.01, ***p <0.001, ****p <0.0001, based on Student’s t test.

**Fig. 7:**
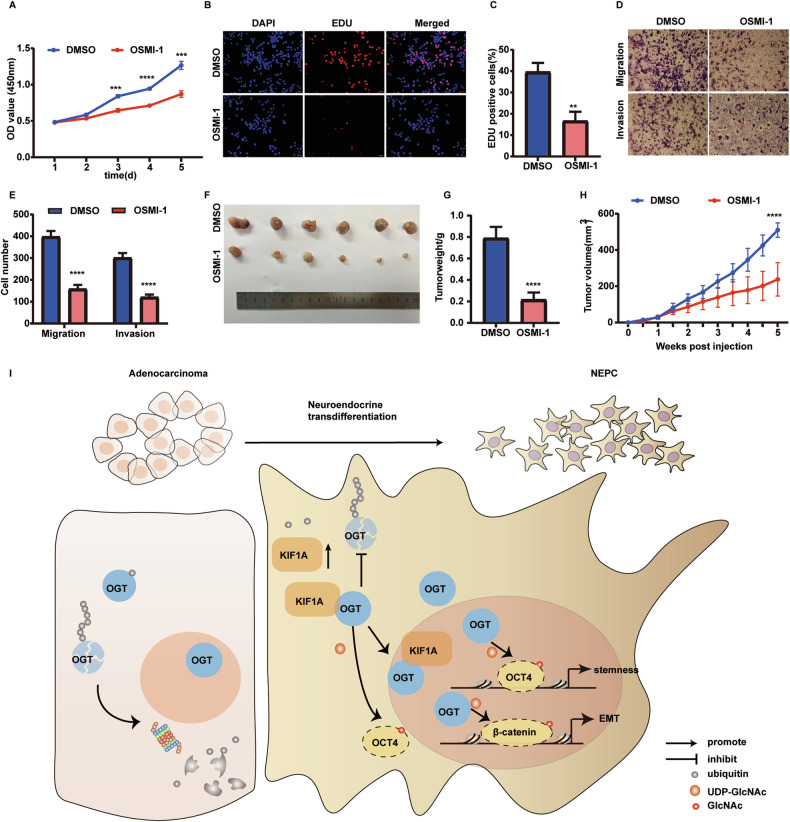
OSMI-1 inhibits aggressive growth of NE transdifferentiated PCa cell in vitro and in vivo. **A** Cell viability assessed by CCK-8 assay treated with 20 μM OSMI-1 or an equivalent dose of DMSO at different time points in LNCaP-AI cells. **B**, **C** Representative images and quantification of EDU assay in indicated LNCaP-AI cells with 20 μM OSMI-1 or an equivalent dose of DMSO for 72 h. **D**, **E** Representative images and quantification of transwell migration and matrigel invasion assays in indicated LNCaP-AI cells. **F**–**H** Effect of OSMI-1 on tumorigenesis in vivo evaluated with xenografts model. LNCaP-AI cells were subcutaneously injected into nude mice and the nude mice were randomly divided into control (DMSO), OSMI-1 (1 mg/kg, intravenously) after 7 days. Each group was intravenously administered every other day for 4 weeks. Representative images of xenograft tumors(**F**), Tumor weight (**G**), The growth curve (**H**). **I** Proposed model for KIF1A in promoting development of NEPC. Increased KIF1A forms complex with OGT escaping ubiquitin-proteasome mediated degradation to enhance OGT stabilization and nucleus accumulation. Moreover, intranuclear OGT regulates OCT4 and β-catenin O-GlcNAcylation to promote EMT and stemness, ultimately facilitates NE transdifferentiation to NEPC induced by KIF1A.

To further validate the role of OGT in the NE transdifferentiation induced by KIF1A, we overexpressed OGT in KIF1A depleted LNCaP-AI cells. KIF1A knockdown resulted in decreased expression of NCAM1, ENO2 and SYP, while OGT overexpression restored the decreased expression of these NE markers (Fig. 6A). OGT overexpression abolished the effect of KIF1A knockdown, including the upregulation of E-cadherin and the downregulation of Vimentin and N-cadherin (Fig. 6B). Similarly, depletion of KIF1A in LNCaP-AI cells elicited decreased expression of stemness related genes and sphere formation ability, while OGT overexpression restored these capacities (Fig. 6C–E). Moreover, the decreased capability of cell proliferation, migration and invasion caused by KIF1A knockdown significantly increased with OGT overexpression (Fig. 6F–J). Overexpression of OGT also rescued the decreased tumor xenografts growth of LNCaP-AI cells induced by shKIF1A (Fig. 6K–N). The decreased Ki67 index and NE marker expression caused by KIF1A knockdown significantly increased with OGT overexpression (Fig. 6O). Moreover, OSMI-1 treatment inhibited cell proliferation, migration invasion and tumor growth in LNCaP-AI cells (Fig. 7A–H). However, the use of OSMI-1 in LNCaP-AI cells did not affect the AR signaling pathway, neither in terms of AR protein expression nor in the transcription of key downstream molecules (Supplementary Fig. 10A, B). These data suggested that OGT was essential for KIF1A induced NE transdifferentiation and aggressive growth. Collectively, we proposed that KIF1A facilitated NE transdifferentiation of prostatic adenocarcinoma via modulation of OGT and O-GlcNAcylation of OCT4 and β-catenin (Fig. 7I).

## Discussion

In this study, we found that the aberrant expression of KIF1A promotes aggressive phenotypes in PCa, including enhanced cell proliferation, metastasis, colony formation and NE-differentiated competency. We demonstrate that KIF1A promotes NE phenotype in vivo and in vitro cell line models and hypothesize important pathogenic role of KIF1A in NEPC. Previous studies have proven the critical role of KIF1A in the development of the nervous system, which powers the movement of nuclei in differentiating brain stem cells and transports synaptic precursors and dense core vesicles in axons [18, 29]. KIF1A dysfunction leads to severe neurodevelopmental and neurodegenerative diseases in human, such as optic nerve atrophy [30]. KIF1A promotion of NE transdifferentiation in PCa may be related to its critical role in neural development, which needs to be further explored. Admittedly, our findings are primarily based on in vivo and in vitro cell line models. Further validation in pre-clinical PCa organoid models and GEMMs are warranted.

OGT is the only known O-linked N-acetylglucosaminyl transferase primarily located in the cytoplasm and nucleus [27, 31]. It is aberrantly expressed in many cancers and contributes to several hallmarks of cancer, ranging from invasion and metastasis, evading apoptosis, sustained angiogenesis to epigenetic reprogramming [26, 28, 32–36]. Notably, overexpression of OGT is associated with poor prognosis in PCa [37]. OGT knockdown inhibits cell proliferation, migration, and invasion in PCa cell lines including PC3, LNCaP and C4-2B [38–40]. OSMI-1 increases the sensitivity of PCa cells PC3 to doxorubicin [41]. These findings support the potential value of OGT in advanced PCa. In this study, OGT overexpression reverses KIF1A-knockdown related phenotypes in LNCaP-AI cells, adding its potential role in PCa development to previous findings. Here, we found that KIF1A promotes nuclear translocation of OGT and inhibits its cytoplasmic ubiquitination, which is consistent with the reduced ubiquitination of OGT after TET3 recruits OGT into the nucleus [42]. Interestingly, immunohistochemistry shows that the levels of OGT and whole protein O-GlcNAcylation are upregulated in NEPC compared to adenocarcinoma, while OGT mRNA levels are not statistically different in NE-related groups versus control in NEPC datasets, suggesting the upregulation of OGT in NEPC is induced by enhanced protein stabilization rather than transcriptome alteration.

Accumulating evidences suggest that O-GlcNAcylation is associated with NE feature. Overexpression of OGT promotes self-renewal, differentiation and reprogramming efficiency of mouse embryonic fibroblasts via O-GlcNAcylation of the Yamanaka factors (c-myc, OCT4, SOX2, and KLF4) [43], while MUC1-C regulates lineage plasticity driving progression to NEPC via mediating the same Yamanaka factors [10]. It is plausible that increased O-GlcNAcylation promotes lineage plasticity through Yamanaka factors. OGT regulates β-catenin O-GlcNAcylation and facilitates EMT in liver, colorectal and pancreatic cancer [44–46]. OGT also promotes O-GlcNAcylation of the key components of the PRC1 (BMI1 and RING1B) and PRC2 (EZH2), which are important for stem cell-like phenotype and treatment resistance [40].

Many transcriptional regulator or epigenetic regulators have been associated with acquisition of lineage plasticity in PCa [8–14, 47]. However, most of these drivers are not currently feasible as pharmacological targets. We identified O-GlcNAcylation of these drivers in PCa with NE phenotype, suggesting the potential to utilize OGT inhibitors in this subset of PCa patients. The limitation of this study lies in the conclusions drawn from the use of (i) single cell line (LNCaP-AI) and (ii) xenograft models. Further assessment of efficacy of OSMI-1 in more sophisticated models, such as patients derived organoids or genetically engineered mouse models (GEMMs) would provide a more comprehensive evaluation of OGT inhibitors in the context of NEPC.

In this study, we elucidated a positive association between KIF1A expression and NE phenotype. Our findings indicated that KIF1A facilitates NE differentiation by regulating the OGT-mediated O-GlcNAcylation. Furthermore, targeting O-GlcNAcylation presents a promising therapeutic strategy to impede the development of NEPC.

## Supplementary information

Supplementary Materials and Methods

Supplementary tables

Supplementary Figures and Legends

Orignal Western blots

## Data Availability

All data and materials during the current study are available from the corresponding author on reasonable request.
