# Supplementary material for: KIF1A promotes neuroendocrine differentiation in prostate cancer by regulating the OGT-mediated O-GlcNAcylation
Source: Cell Death Dis. 2024 Nov 6;15(11):796. doi: 10.1038/s41419-024-07142-2 (PMC11542072; doi:10.1038/s41419-024-07142-2)
Supplement: Supplementary file 1 — Supplementary Materials and Methods [file 41419_2024_7142_MOESM1_ESM.docx]

**Supplementary Materials and Methods**

**Immunohistochemistry**

PV9000 kit (Zsbio) was used to detect protein expression on 4μm paraffin sections. After dewaxing and hydration, sections were performed antigen retrieving in EDTA (pH 9.0) for 10 min (for OGT detecting) and citrate for 10min (for O-GlcNAcylation detecting) in a pressure cooker. The sections were incubated with 3% H2O2 for 10 minutes at room temperature to block endogenous. Nonspecific antibody binding was blocked by incubating with goat serum (ZLI-9056; Zsbio) for 30 min at room temperature. Slides were then incubated with anti-OGT (1:50, cat no.177941; Abcam), anti- O-GlcNAcylation (1:200, cat no.MA1-0721; Invitrogen) at 4 °C overnight. Slides were then washed and incubated with polymer helper for 20 min and polyperoxidase for 30 min. The sections were developed with the diaminobenzidine substrate kit (ZLI-9018; Zsbio) and observed under microscope.

Sections were evaluated by a pathologist in a blinded fashion. The overall result in each case was classified as negative if less than 10% of the tumor area was stained, and as positive if 10% to 100% of the tumor area was stained. In addition, the percentage of positive tumor cells on each section (10%-100%) was multiplied by the predominant staining intensity pattern (1, weak; 2, moderate; 3, strong); the total score could be up to 300 H points.

**RNA isolation and quantitative real‑time PCR analysis**

Total RNA was extracted using the TRIzol reagent (Thermo Fisher Scientific, USA) and reverse-transcribed with cDNA Reverse Transcription Kit (Toyobo, Japan) according to the manufacturer’s protocols. Quantitative real‑time PCR was performed using the SYBR Green mix (BIO-RAD, USA). Primers used for qRT-PCR were listed in **Supplementary Table 2**.

**Immunoprecipitation (IP), Co-Immunoprecipitation (Co-IP) and Western blot**

Western blot, Co-IP and IP assays were performed as previously described [24]. To determine the subcellular localization of OGT, nuclear and cytoplasmic protein was extracted using the Nuclear and Cytoplasmic Protein Extraction Kit (Beyotime Biotechnology, China) following the manufacturer’s instructions. The detailed information of antibodies was summarized in **Supplementary Table 3**.

**Cell proliferation, migration, invasion and colony formation analysis**Cellular proliferation capacitywas measured by Cell-Light™ EdU DNA Cell Proliferation (EdU) assays (Ribobio, China) and cck8(Ribobio, China) according to the manufacturer’s protocol. The transwell assay was used to measure the migration and invasion of PCa cells. For clonal experiments, cells were seeded at low density (500 cells/well) in a 6-well plate and allowed to grow until visible colonies appeared. Clones were counted within 2 weeks.

**Sphere formation assay**

For sphere formation assay, 3000 single cells/well were cultured in serum-free 1640 (Gibco, USA) supplemented with 2% B27 supplement (Thermo Fisher Scientific,USA), 20 ng/ml EGF (Yeasen Biotechnology (Shanghai), China) and 10 ng/ml bFGF (Yeasen Biotechnology (Shanghai), China) in ultralow attachment plate. Spheroids with diameter > 75 μm were counted.

**Immunofluorescence (IF)**

Cells were grown on glass coverslips in 24-well culture dishes and fixed with 4% paraformaldehyde for 30 min. After washing with 1×PBS, the cells were permeabilized with 0.1% Triton X-100 in 1× PBS for 10 min and blocked with 5% goat serum for 1 h at room temperature. The cells were then incubated with primary antibodies at 4 °C overnight. The detailed information of primary antibody was summarized in Supplementary Table 3. After washing with 1×PBS for 3 times, the cells were incubation with goat anti-mouse Alexa Fluor 647 and goat anti-rabbit Alexa Fluor 488 (Beyotime Biotechnology, China). Images were processed under a confocal microscope (OLYMPUS, Japan).
