# Supplementary material for: KIF1A promotes neuroendocrine differentiation in prostate cancer by regulating the OGT-mediated O-GlcNAcylation
Source: Cell Death Dis. 2024 Nov 6;15(11):796. doi: 10.1038/s41419-024-07142-2 (PMC11542072; doi:10.1038/s41419-024-07142-2)
Supplement: Supplementary file 2 — Supplementary tables [file 41419_2024_7142_MOESM2_ESM.docx]

| **Supplementary Table 1. siRNAs and ShRNA used in this study** | |
| --- | --- |
| siRNA | Sequence |
| siKIF1A-1 | GACCCAACAAGAACAAGAATTUUCUUGUUCUUGUUGGGUCTT |
| sKIF1A-2 | CAGCAUCUCUGCCGAAUAUTTAUAUUCGGCAGAGAUGCUGTT |
| shKIF1A | ACCGGTGACCCAACAAGAACAAGAATTCAAGAGATTCTTGTTCTTGTTGGGTCTTTTTTGAATTC |
| These siRNA were purchased from GenePharma (Shanghai, China). | |

| **Supplementary Table 2. Primers used in this study** | | |
| --- | --- | --- |
| Gene | Forward | Reverse |
| PSA | GCCTGGATCTGAGAGAGATATCATC | ACACCTTTTTTTTTCTGGATTGTTG |
| TMPRSS2 | GGTAAACTCTCCCTGCCACA | TACTCCAGGAAGTGGGGATG |
| KIF1A | TGATGGGCAAGCAGGAGAAG | TTGCCCTTGTTCTTGGGGTT |
| OGT | CGGCTGCGTGTAGGATATGT | GCCATCACCTTCACTCGGAA |

| **Supplementary Table 3. Antibodies used in this study.** | | |
| --- | --- | --- |
| **Antibody** | **Cat no.** | **Application** |
| GAPDH | cat no. ab181602; Abcam | WB(1:5000) |
| KIF1A  KIF1A | cat no.ab180153; Abcam  cat no. ab91029;Abcam | WB(1:5000), IF(1:100), Co-IP(500ug/ug)  IHC(1:100) |
| OGT | cat no.177941;Abcam | WB(1:1000), IF(1:100), Co-IP(500ug/ug) |
| NSE | cat no. 66150-1-Ig;Proteintech | WB(1:5000) |
| SYP | cat no.17785-1-AP;Proteintech | WB(1:2000) |
| NCAM1 | cat no.ET1702-43;Huabio | WB(1:1000) |
| SOX2 | cat no.ab92494;Abcam | WB(1:1000) |
| BMI1 | cat no.ab126783;Abcam | WB(1:1000) |
| OCT4 | cat no.11263-1-AP;Proteintech | WB(1:1000) |
| β-catenin | cat no.CY3523;Abways | WB(1:1000) |
| E-cadehin | cat no.SC-8426;Santa Cruz | WB(1:200) |
| N-cadehin | cat no.CY5015;Abways | WB(1:500) |
| Vimentin | cat no.CY5134;Abways | WB(1:1000) |
| CD133 | cat no.66666-1-Ig;Proteintech | WB(1:500) |
| O-glcNAcylation | cat no. SC-59623; Santa Cruz | WB(1:200), IP(500ug/ug) |
| O-glcNAcylation | cat no.MA1-0721;Invitrogen | IHC(1:200) |
| LaminA/C | cat no.10298-1-AP;Proteintech | WB(1:5000) |
| H3 | cat no.68345-1-Ig;Proteintech | WB(1:10000) |
| Ubiquitin | cat no.10201-2-AP; Proeteintech | WB(1:1000) |

| **Supplementary Table 4.Up-regulated genes in NE-associated samples compared to controls** | | | | | |
| --- | --- | --- | --- | --- | --- |
| **Genes** | Mean of FKPM | | standard deviation | | Cohen's d |
|  | CRPC(m1) | NEPC(m2) | CRPC(s1) | NEPC(s2) |  |
| KIF1A | 0.0149564 | 3.3184622 | 0.51756 | 2.59398 | 0.94432 |
| TUBB2B | 0.2842989 | 11.040008 | 0.63995 | 10.0055 | 0.214 |
| UBE2C | 0.3939181 | 6.6301745 | 1.92649 | 6.83226 | 0.24751 |
| NUF2 | 0.7610848 | 9.0355237 | 3.84633 | 9.18061 | 0.16703 |
| CDC25B | 2.0423116 | 21.069994 | 3.01248 | 17.6491 | 0.11871 |
| STMN1 | 11.88302 | 67.634019 | 27.2678 | 64.5278 | 0.02272 |
| RGS3 | 0.8728213 | 4.1548955 | 1.80897 | 3.06302 | 0.51872 |
| HJURP | 9.1603725 | 30.815186 | 15.1166 | 25.9556 | 0.048 |
| MLLT11 | 2.6979063 | 7.9169674 | 2.34606 | 7.49855 | 0.16909 |
| UBE2T | 3.1912842 | 8.2286682 | 5.26638 | 5.65179 | 0.16882 |
| Cohen's d=│(m1-m2)/√(s1^2+s2^2)/2│ | | | | | |

| **Supplementary Table 5. Protein combined with KIF1A** | | | | | | |
| --- | --- | --- | --- | --- | --- | --- |
| CLTC | KHSRP | NAPA | PPP6C | ABHD10 | DKFZp686D0638 | RPRD1B |
| MYH9 | MARS1 | RPS5 | AP3M1 | THEM6 | DKFZp451J085 | PSMB6 |
| MYO6 | KPNB1 | PSME3 | RPS3A | RPL8 | DENND10 | NUCB2 |
| DSP | SEC16A | PSMA7 | RPS2 | PNPLA4 | CPSF6 | NT5DC2 |
| SF3B1 | CHP1 | CYB5R3 | DCTN2 | NDUFS8 | C17orf75 | NDUFA5 |
| FLNB | SEC22B | AZGP1 | RFC4 | LASP1 | BUB3 | MRPL4 |
| PSMD2 | RTRAF | STBD1 | EIF3F | AHCY | ATAD1 | MBD3 |
| RRBP1 | PYCR1 | MRPS35 | PGK1 | PSME1 | ARG2 | LSM4 |
| CKAP4 | STRAP | CHCHD3 | RFC2 | MED27 | ADA | KDSR |
| HSPA5 | ECHS1 | PEX11B | PSAT1 | STOML2 | ZC2HC1A | H3-7 |
| CTNNA1 | ACAT1 | DHRS7B | ACTR2 | MLEC | TTC26 | EIF4H |
| EIF3L | HSP90AB1 | ANXA2 | CCDC51 | DCAF7 | TTC19 | DCUN1D1 |
| EPRS1 | HADHB | VTA1 | TALDO1 | STX5 | STK3 | CYB5R1 |
| FLNA | COPS4 | MTARC2 | DDOST | RSU1 | STK25 | COX5B |
| FASN | RUVBL1 | ARPC2 | CAP1 | PSMA3 | SAE1 | COMMD2 |
| MAP1B | SAMM50 | SEPTIN11 | BAG3 | PABPC4 | RP2 | CDC37 |
| DARS1 | PSMD12 | ELAVL1 | TOMM70 | HNRNPC | PTGR1 | C2orf49 |
| IGF2R | PHGDH | DHODH | ALDH3A2 | DHRS7 | PSMC4 | ATP6V1D |
| VCP | DDX5 | derp12 | TKT | DECR1 | MRPS31 | USE1 |
| FOLH1 | CCT2 | RMDN1 | SNTB2 | ARG1 | MCAT | UGT2B17 |
| MME | HSPA1B | PDHB | ARCN1 | MRRF | MBOAT7 | TPM1 |
| HSPD1 | CDK5RAP3 | ECH1 | SYNCRIP | ERCC1 | MANEA | TMEM223 |
| CCT8 | KARS1 | ACADSB | GALNT7 | MRPS2 | IVD | SYBL1 |
| ACTN4 | ATP6V1A | PSMC3 | QSCN6 | SEPTIN7 | IST1 | SLC9A3R1 |
| HSPA8 | SFPQ | SMARCE1 | GPD2 | OLA1 | IDH3G | RTN4 |
| AP2A1 | ACAD9 | RNH1 | LETM1 | MTCH1 | GRTP1 | RRAS |
| HADHA | COPB2 | PTBP1 | HEXB | DLST | GORASP2 | RPP38 |
| HRNR | FOLH1 | SF3A2 | PSMD1 | DDX17 | GALNT4 | PRMT1 |
| DKFZp781K0743 | DRIP4 | IMPDH2 | VPS51 | ATP6AP2 | GALNT2 | PPT1 |
| MYO1D | ATP2A2 | SERPINB3 | CTNND1 | TSG101 | EIF2B4 | PCMT1 |
| DDB1 | AARS1 | KPNA2 | ACLY | DKFZp686P18 | DYNC1LI2 | PAFAH1B3 |
| JUP | COPA | PAICS | IARS1 | UQCRFS1 | DNAJB1 | NDUFB5 |
| ATP5F1A | HSD17B10 | NARS1 | PIK3C2A | SERPINB12 | DDX39B | NCCRP1 |
| COPB1 | SNRNP40 | DLAT | PGAM1 | SCCPDH | COX18 | MSRA |
| ATP5B | MDH2 | RCC2 | YWHAZ | HIBCH | BYSL | MRPL16 |
| SSB | SLC25A11 | ACADVL | PPIA | HADH | ANK3 | MAPRE2 |
| XRCC6 | ETFA | COG6 | NIPSNAP3A | PDIA6 | ABI1 | IYD |
| SF3A1 | ILF2 | TGM3 | RPL10A | GPS1 | TXLNA | GTF2F2 |
| IMMT | DRG1 | SEL1L | ATP6V1E1 | CPSF7 | TTC30A | GIPC1 |
| XRCC5 | MRPS27 | NELFB | HNRNPA1 | CPNE3 | TRMT10C | GET4 |
| ATP2B1 | PTGES2 | COPG1 | AIMP2 | CNP | SF4 | FLJ22222 |
| EEF2 | CLTB | SART3 | COPS6 | CANT1 | SEC23B | EMG1 |
| FLOT1 | UQCRC2 | DSC1 | ERP29 | SCARB2 | RCC1 | DHRS4 |
| RTCB | BAF53A | VPS35 | PHB2 | SARG | RBM4B | COA7 |
| MAOA | EIF2S3 | UBA1 | LRRC59 | MTX1 | POMGNT2 | CMBL |
| ALB | NONO | GCS1 | RPS4X | MTA2 | PLAA | CLPP |
| HSP90B1 | EPHX1 | VPS53 | RPL7A | LANCL1 | PIGT | CHMP1B |
| MAP4 | RDX | NCAM2 | EIF2B2 | HSD17B4 | PDK3 | CERS2 |
| ATXN2L | RPN2 | EMC1 | APEX1 | FUS | PARVA | CD63 |
| TLN1 | EEF1G | CAPRIN1 | LDHB | CTTN | ND4 | ARFIP2 |
| PHB2 | TKFC | ATXN2 | SERPINB6 | ATP6V0D1 | MUT | TREX1 |
| PABPC1 | RBBP4 | AFG3L2 | WDR82 | PTCD3 | MARK2 | TP53RK |
| EIF3E | NDUFS1 | OGDH | RPL7 | PAIP1 | IRF2BPL | TMEM106 |
| PKM | TCP1 | COBLL1 | MRPS22 | DBT | EMC10 | TIMM29 |
| EIF3B | COG2 | KIF5B-RET | LDHA | CARS2 | EIF3D | THOC6 |
| CSE1L | RBM14 | COG1 | EIF4A3 | STEAP2 | EIF2B3 | SSR1 |
| FKBP4 | UFL1 | MIA3 | CYB5R1 | CLINT1 | DNAJC3 | SPC24 |
| P4HB | LIMA1 | SPTBN2 | STUB1 | TFRC | DKFZp586J151 | SNC73 |
| RARS1 | TUBGCP2 | PLEC | RPL4 | MTPAP | DCP1A | SLC25A17 |
| NSF | LARS1 | CSTA | RPL3 | GLB1 | BYSL | SDCBP2 |
| SF3B3 | PRPF8 | PAM16 | CTSD | GFPT1 | ATL3 | RPP30 |
| UTRN | RAB7A | SNRPD2 | TUFM | GBA1 | ASCC1 | PKP3 |
| RFC5 | APOOL | RPS16 | SRP54 | FUT8 | ALCAM | PECR |
| PSMD11 | RPLP0 | MYL12B | MRPS9 | CSDE1 | SRPR | MBLAC2 |
| CCT4 | AIDA | YWHAG | STAU1 | ADD1 | SPTLC1 | KPNA4 |
| SRP68 | ATP5PB | RAB14 | SEPT9 | TUBGCP4 | SAMHD1 | HSDL1 |
| ATP1A1 | PPP2CA | RPS12 | PRPF31 | PYGB | REPS1 | HM13 |
| eIF3a | SKIC8 | TUBA1C | ICAM1 | PKP1 | POU4F3 | GNPDA1 |
| KIF1A | PSMD7 | RPS11 | BAIAP2 | NCBP1 | PLBD2 | DNAJC8 |
| YWHAE | PSMA1 | ARMC10 | SUN2 | ITGB1 | PGM1 | DKFZp762 |
| AIMP1 | RPL5 | MANF | MTDH | EIF3C | NSUN2 | C3orf33 |
| EIF3I | ETFB | AP2S1 | HSD17B4 | SEC31L1 | ME2 | ARMCX3 |
| HEL-215 | G3BP2 | RPS14 | PFKP | GARS1 | LARP4 | APOL3 |
| RACK1 | PUF60 | MRPS23 | CARS1 | COG5 | LACTB | XXYLT1 |
| EIF3M | G3BP | VDAC3 | ANXA6 | DCTN1 | KDM1A | USP14 |
| DDX6 | SELENBP1 | PSMB4 | SACM1L | BAG6 | INTS7 | UQCRFS1 |
| RUVBL2 | FH | TMED10 | COG8 | ARID1A | GLUD1 | TWF1 |
| TOM1L2 | ACTR3 | PLGRKT | XPO1 | TUBB | GATAD2A | SUCLG2 |
| SLC25A24 | SERBP1 | NPM1 | FAM120A | VDAC1 | FHIP2A | SUCLG1 |
| YARS1 | GPI | GGCT | USO1 | SF3B5 | DNAJC10 | SNX27 |
| PDIA4 | DARS | PDCD10 | RPLP2 | TIMM13 | DLGAP4 | SERPINB7 |
| DDX3X | DDX1 | RPSA | LYZ | RPL38 | CUL4B | SBDS |
| NCL | ADAM9 | GSTK1 | CAPZA1 | SNRPD3 | CUL1 | QPCTL |
| HNRNPM | ZW10 | CDK1 | S100A7 | ELOC | CLPB | QKI |
| FOLH1 | EXOC3 | HSD17B8 | EEF1E1 | H4C1 | CD2AP | PRSS8 |
| KIF5B | PRPF6 | CKB | CISD2 | CYB5A | C9orf88 | PRKAR1A |
| DSG1 | NCKAP1 | SET | EIF4E2 | SRP9 | ATP5MF-PTCD1 | POLDIP3 |
| EIF4G1 | IARS1 | CBX3 | RPS8 | DCD | ASS1 | NT5C3A |
| ATP5PD | FLG2 | BNIP1 | CBR4 | YWHAB | AP3D1 | MTX3 |
| HNRNPA2B1 | DOCK7 | PYCR3 | FAM162A | ISOC2 | XPO7 | MRPL45 |
| GAPDH | PRDX1 | EIF4E | EIF5A | PTRH2 | WASHC5 | MRI1 |
| ALDOA | RPS19 | ERGIC1 | COX4I1 | SNRPD1 | TFIP11 | MAT2A |
| EIF2S1 | REEP3 | TFG | PYCR2 | RPL30 | TBCD | KPNA3 |
| DAP3 | SNRPA | NDUFV2 | SEC13 | CRIP2 | SF3B2 | INTS12 |
| ENO1 | PBDC1 | HIBADH | GOSR1 | YBX1 | RALGAPB | HSDL2 |
| FLOT2 | BAG2 | HEL-S-95n | hEMMPRIN | PIP | PRPF40A | GTF2F1 |
| PSMD6 | NDUFS3 | PSMB5 | COPS7A | LOC392793 | PPP1R10 | GOT1 |
| HSP90AA1 | TPI1 | MTIF3 | TOLLIP | PSMA2 | OGT | FLOT2 |
| CCT7 | COPE | MTCH2 | RDH11 | PCBP1 | DDX46 | F11R |
| RPA1 | TMX2 | HNRNPH1 | PSMA6 | HMGB1 | CHERP | ESD |
| FAM98B | CIAO2B | RANGAP1 | GOLM1 | YWHAH | A2ML1 | DPM1 |
| OCIAD1 | UBE2N | PPP3CA | FUBP1 | SNRPB2 | NUP210 | BTF3L4 |
| EPCAM | SOD1 | PAK2 | EWSR1 | RPL17 | LARP4B | TMEM205 |
| EIF2B1 | RALA | OXA1L | DNAJA3 | MPC2 | EDC4 | RPL34 |
| BDH1 | MRPS6 | MARK2 | CS | ANP32A | COL1A2 | NME3 |
| ST3GAL1 | HMGB2 | IFT74 | CKMT1ACKM | TPT1 | NDUFAF3 | COX5A |
| RPL6 | H2BC21 | GALNT3 | ATXN10 | DNAJC19 | NACA | UBL4A |
| PSMC5 | GATC | DARS2 | RCN1 | SSBP1 | MPG | TOMM40 |
| FTH1 | COPS8 | ABCE1 | PSMC2 | SPCS2 | CFAP20 | RAB27A |
| ERLIN2 | ATP6V1G1 | VPS33A | KHDRBS1 | RPL18 | AMZ2 | JAGN1 |
| AP1B1 | UBE2L3 | SLC44A2 | HNRNPA3 | NDUFB4 | YIF1B | HIST1H1E |
| MRPL44 | TMCO1 | OMA1 | ETF1 | MRPL50 | RPL19 | SDF2L1 |
| RBMX | SEC11A | MYH14 | ENTPD2 | MGST1 | RAB21 | NTPCR |
| IDH1 | RPL31 | HSPA9 | CDC73 | MCTS1 | LSM12 | H1-5 |
| ANXA5 | PSMD9 | GHDC | APMAP | DENR | FKBP11 | CAPNS1 |
| AGK | POLR2H | FARSA | SIL1 | EDF1 | EIF3J | BLOC1S4 |
| ACTN1 | PFN2 | EXOC5 | PIGS | DCXR | CHMP5 | AP1S1 |
| TXNDC5 | NDUFB6 | CSTF3 | MAPK1 | RPL24 | AP3S2 | TMED7 |
| TOR1AIP1 | MRPL49 | VCL | MAGT1 | RPL23A | PPIH | SPCS3 |
| RDH13 | CBX1 | VAC14 | LPCAT3 | RPL10 | MSN | RALB |
| PSMD8 | TRIR | STT3A | LNPK | COPZ1 | MIF4GD | RAB23 |
| GGH | RPS23 | SCYL1 | HDAC1 | RPS25 | CCDC124 | MAGOH |
| CLTA | RPL29 | NCSTN | FAAH | PRDX6 | CACYBP | H1-5 |
| BPNT2 | METTL7A | LRRN1 | DYNC1LI1 | SCAMP1 | ALDOC | ELOB |
| SNRPB | JPT2 | IFT88 | CNOT2 | HNRNPA0 | SARNP | CHCHD6 |
| SLC25A1 | GNAI3 | CUL5 | ATP6V1C1 | H1-10 | PSMB2 | CBR1 |
| PSMC6 | FAM210B | TJP1 | RO60 | GID8 | NAP1L1 | ARL2 |
| CSNK1A1 | CHID1 | PRR36 | NAGLU | EIF6 | EMC8 | TIMM50 |
| FDFT1 | ARF6 | LARP1 | AHCYL1 | CALML5 | CHMP4B | CCNK |
| TOM1 | TPD52L1 | HDLBP | SHTN1 | RPL28 | ATAD3A | ACADS |
| SQOR | TMED3 | GTF2I | SGPL1 | PDAP1 | ARHGDIA | SPINT1 |
| UQCRC1 | TAGLN2 | GOLGA3 | HSPA14 | DCTN3 | AK4 | SEC61A1 |
| TRIM21 | SYNJ2BP | GNAS | GUSB | VPS37B | UQCC1 | SDHA |
| TMEM43 | SFT2D3 | CYFIP2 | GSS | SBSN | TOMM34 |  |
| ATP6V1B2 | RPL11 | RAD50 | CPT2 | RPS7 | SNU13 |  |
| PACSIN2 | PHF5A | FLG | COG7 | NDUFAF4 | SHINC3 |  |
| NDUFV1 | MRPL10 | FLNB | CDC23 | LCN1 | NUDT5 |  |
| EIF2A | MORF4L1 | CNOT1 | BAIAP2L1 | FKBP3 | MRPL46 |  |
| SHMT2 | FABP5 | TUBB2C | ASNS | TUBB6 | KPNA6 |  |
| GOLM2 | DRAP1 | ATP1B3 | STAT1 | TMED1 | KPNA1 |  |
| SLC3A2 | CST6 | TUBB4A | RANBP9 | SYBL1 | HAUS1 |  |
| CANX | CSRP1 | MYL6 | NUMB | RPL13 | GM2A |  |
| VPS45 | CDK2 | keratin | LTF | PURA | GATD3B |  |
| FARSB | BRI3BP | UQCR10 | HNRNPU | HEL-S-39 | FUNDC2 |  |
| SRP72 | BLOC1S1 | VAPB | EXOC7 | GSTM3 | EXOSC4 |  |
| COG4 | NDUFS7 | PEX16 | DDX41 | EMC3 | CNDP2 |  |
| APEH | NAP1L4 | PPP1CA | ARMC8 | CAB39L | 44M2.1 |  |
| ALDH6A1 | TMPO | BTF3 | MRE11 | SCAMP3 | RPS6 |  |
| TMEM214 | SYNGR2 | CSTB | UPF1 | RAB11A | PSMD14 |  |
| STIM1 | SOD2 | VIL2 | MYO1B | PPA2 | PPA1 |  |
| MYH10 | SNX5 | PPP1CC | MED24 | FAM98A | MED8 |  |
| EXOC1 | SELS | PPP1CB | LARS1 | EMD | MED4 |  |
| CAND1 | SDSL | HMGA1 | HIP1 | DCUN1D5 | LYPLAL1 |  |
| SEC31A | RPL36A | MCRIP1 | DNM2 | TRAPPC5 | DNAJC9 |  |
| KTN1 | PRDX5 | SPRR2D | FOCAD | ST13P4 | DNAJA1 |  |
| DHX9 | PEX19 | SLC25A5 | PRKDC | RPL26 | CCNC |  |
| SNRNP200 | PEBP1 | LAMTOR3 | RPL23 | RAB25 | TSFM |  |
| ACTG1 | PAFAH1B2 | ATP5PF | HSPE1 | DECR2 | TAF9B |  |
| SRPRB | ORMDL3 | SERF2 | ATP5MK | COMT | SDHB |  |
| RAB1B | NDUFA6 | ARPC3 | NME1-NME2 | CASP14 | RRAGA |  |
| S100A9 | NCBP2 | ARF5 | RAB8A | PCNA | PSMD4 |  |
| PFN1 | MRPL22 | RPS21 | PIN4 | MTARC1 | MRPL39 |  |
| RAB5C | COMMD9 | NOMO2 | MIEF1 | GSTO1 | GRSF1 |  |
| SRP14 | CNBP | LIN7A | SEC61B | DNAJB11 | GDAP1 |  |
| RPL12 | BLVRB | MRPS25 | VDAC2 | ATP1B1 | EXOSC2 |  |
| S100A8 | ANP32E | MMGT1 | RPS27A | ARFIP1 | ECI2 |  |
| CAPZA2 | AK1 | COX6C | NCBP2AS2 | UFD1 | BLVRA |  |
| VAMP8 | ACAA1 | DAD1 | ARF3 | TCEA1 | WIPF2 |  |
| PYM1 | VPS29 | CISD1 | HMGN1 | SLC25A22 | WDR5 |  |
| EEF1A2 | TMEM9 | SELENOT | RAB5B | SCO2 | TMX1 |  |
| YWHAQ | RPL14 | DYNLL2 | CYCS | PSMB7 | TARDBP |  |
| RAB35 | RHOC | RPS27 | ARF4 | HNRNPAB | SURF4 |  |
| PSMB3 | REEP6 | C18orf32 | CFL1 | GRHPR | NIPSNAP2 | |
| PCNP | PEX11G | NDUFA7 | SF3B6 | ASAH1 | IDH3B |  |
| NDUFB10 | MRPL58 | MRPL14 | RAB10 | SPINT2 | COPS3 |  |
| EIF3K | MRPL24 | COX6B1 | TMED4 | PRKRA | CHMP2A |  |
| RPS18 | HNRNPF | TRMT112 | RPL37A | NUDC | CD9 |  |
| RPS13 | CCDC115 | NDUFA4 | ARPC5L | FIBP | BLMH |  |
| MRPL43 | CAMLG | MCRIP2 | YBX3 | EZR | TRUB1 |  |
| CIRBP | VPS26C | RPS26 | RPL27 | ECI1 | TOM1L1 |  |
| ALYREF | TSN | RPL36 | MZT2B | PSMA4 | TAMM41 |  |
| SFXN3 | PSMD10 | MAP2 | EIF4A2 | NPTN | SNX6 |  |
| RPS9 | PABPN1 | UQCC2 | PDCD6 | NIT2 | RBM17 |  |
| TPD52L2 | NDUFA8 | TP53I11 | COA3 | MAVS | GTF2B |  |
| RPS10 | MRPS28 | RPL32 | TPD52 | GMPPB | GPN1 |  |
| RPL9RPL9P7RP | MRPL17 | L27a | RPL35 | EIF2S2 | SH3GL1 |  |
| NIPSNAP1 | MARCKSL1 | CDC42 | RPL22L1 | CSNK2A1 | PLXDC2 |  |
| FAM3C | MAIP1 | TIMM8B | POLR2E | POLDIP2 | NCEH1 |  |
| NQO1 | GEMIN2 | RPS20 | IKBIP | HRMT1L2 | LMNA |  |
| ARPC4 | DDRGK1 | RPL35A | EMC7 | TPM2 | HAUS4 |  |
| RPL15 | CNOT8 | PPIL1 | YA61 | TIMM44 | USP39 |  |
| RAN | CNOT10 | NDUFA2 | SSR4 | OXCT | TUBG1 |  |
| NAA10 | CCDC134 | LGALS7LGAL | SNRPF | NAPG | TIMMDC1 | |
| MRPS26 | ZFPL1 | IFT27 | SEC11C | MDH1 | SEC23A |  |
| HNRPD | WBP2 | DEFA1DEFA | RPUSD3 | APOL2 | RMND1 |  |
| STX12 | VTI1B | COX7A2L | NDUFC2 | YWHAE/FAM22 | NOB1 |  |
| PSMB1 | TMEM126A | ARPC5 | C7orf50 | PDHA1 | MRPL38 |  |
| VPS28 | TK2 | TBCA | SNAPIN | MRPL1 | MED16 |  |
| PRDX4 | TBC1D7 | RBM3 | NDUFAF2 | METTL15 | LAMP1 |  |
| PRDX3 | STX4 | NDUFS5 | COX20 | MCU | DERPC |  |
