# Supplementary material for: KIF1A promotes neuroendocrine differentiation in prostate cancer by regulating the OGT-mediated O-GlcNAcylation
Source: Cell Death Dis. 2024 Nov 6;15(11):796. doi: 10.1038/s41419-024-07142-2 (PMC11542072; doi:10.1038/s41419-024-07142-2)
Supplement: Supplementary file 3 — Supplementary Figures and Legends [file 41419_2024_7142_MOESM3_ESM.docx]

**Supplementary Figures and Legends**


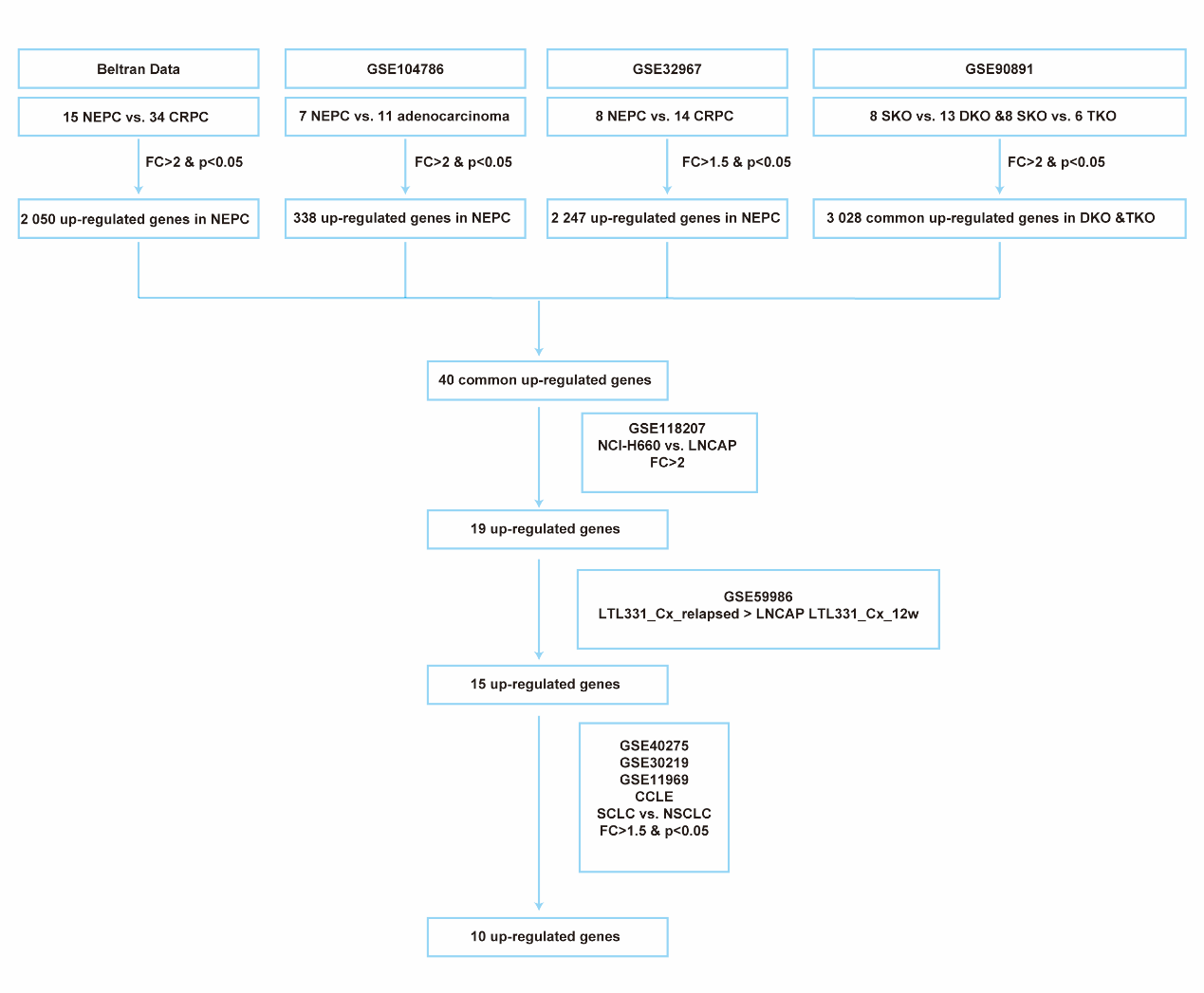


**Supplementary Figure 1. Schematic diagram of dataset integration and the pipeline for putative driver gene selection.** Wilcoxon test was used to calculate p-value in every comparison. Details of the datasets are given in the Methods section, and the screening criteria for each dataset was described in Figure. Finally, a total of 10 candidate genes that were significantly up-regulated in NETs compared to non-NETs. NETs, neuroendocrine tumors; NEPC: neuroendocrine prostate cancer; CRPC, castration-resistant prostate cancer; FC, fold change; SKO, single knockout; DKO, double knockout; TKO, triple knockout.


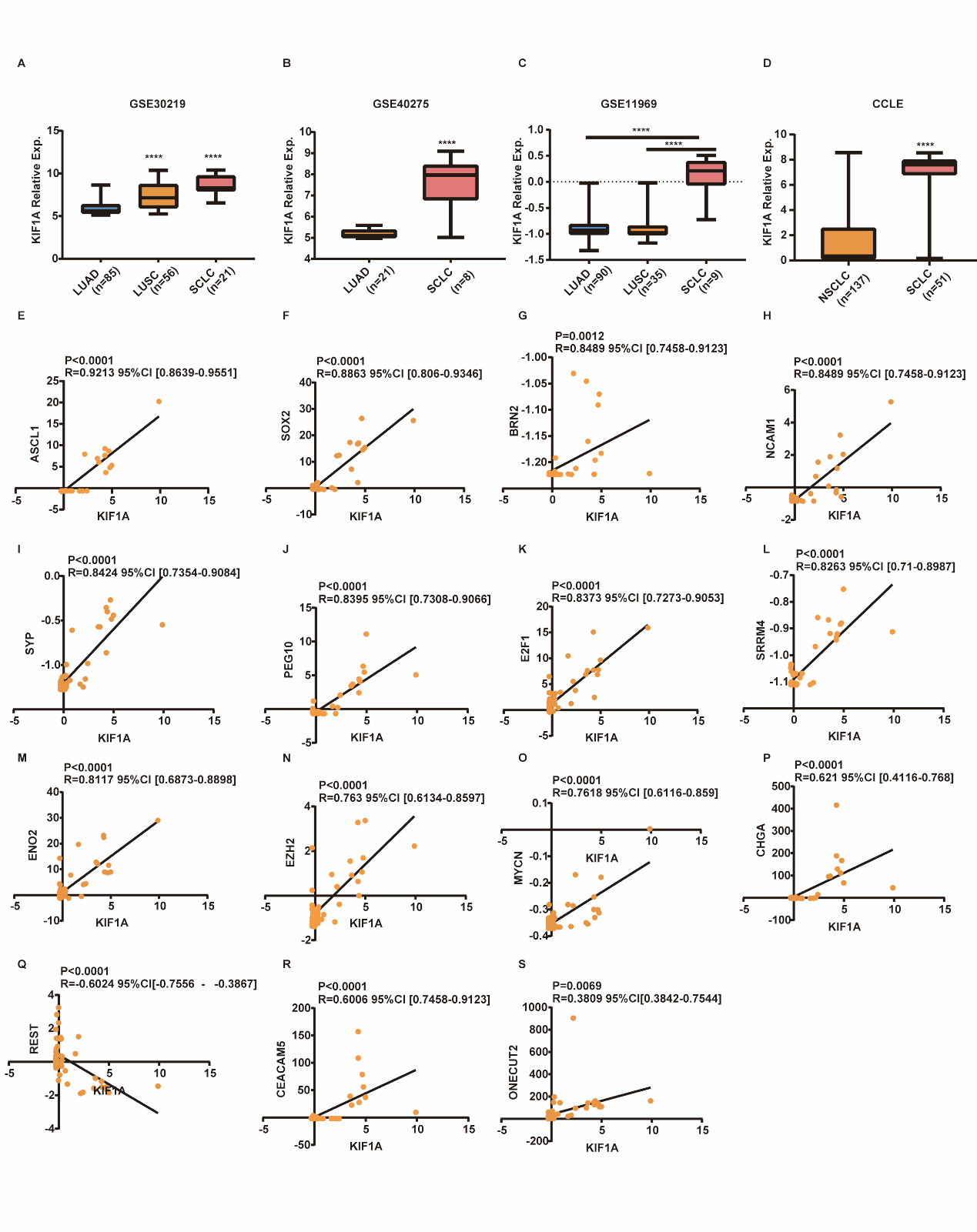


**Supplementary Figure 2. Correlation analysis of KIF1A with NE features.**

A-C. Expression of *KIF1A* in SCLC tissues compared with LUAD or LUSC tissues in GSE42075, GSE30219 and GSE11969 dataset. D. The expressions of KIF1A in SCLC cell lines compared with NSCLC cell lines in CCLE dataset. E-S. The correlation analysis between KIF1A and NE-related genes expressions in Beltran 2016 dataset. Pearson's r correlation coefficient was calculated to show the correlation. **** p<0.0001, based on Student’s t test. SCLC, small cell lung cancer; LUAD, lung adenocarcinoma; LUSC, lung squamous carcinoma; NSCLC, non-small cell lung cancer;.


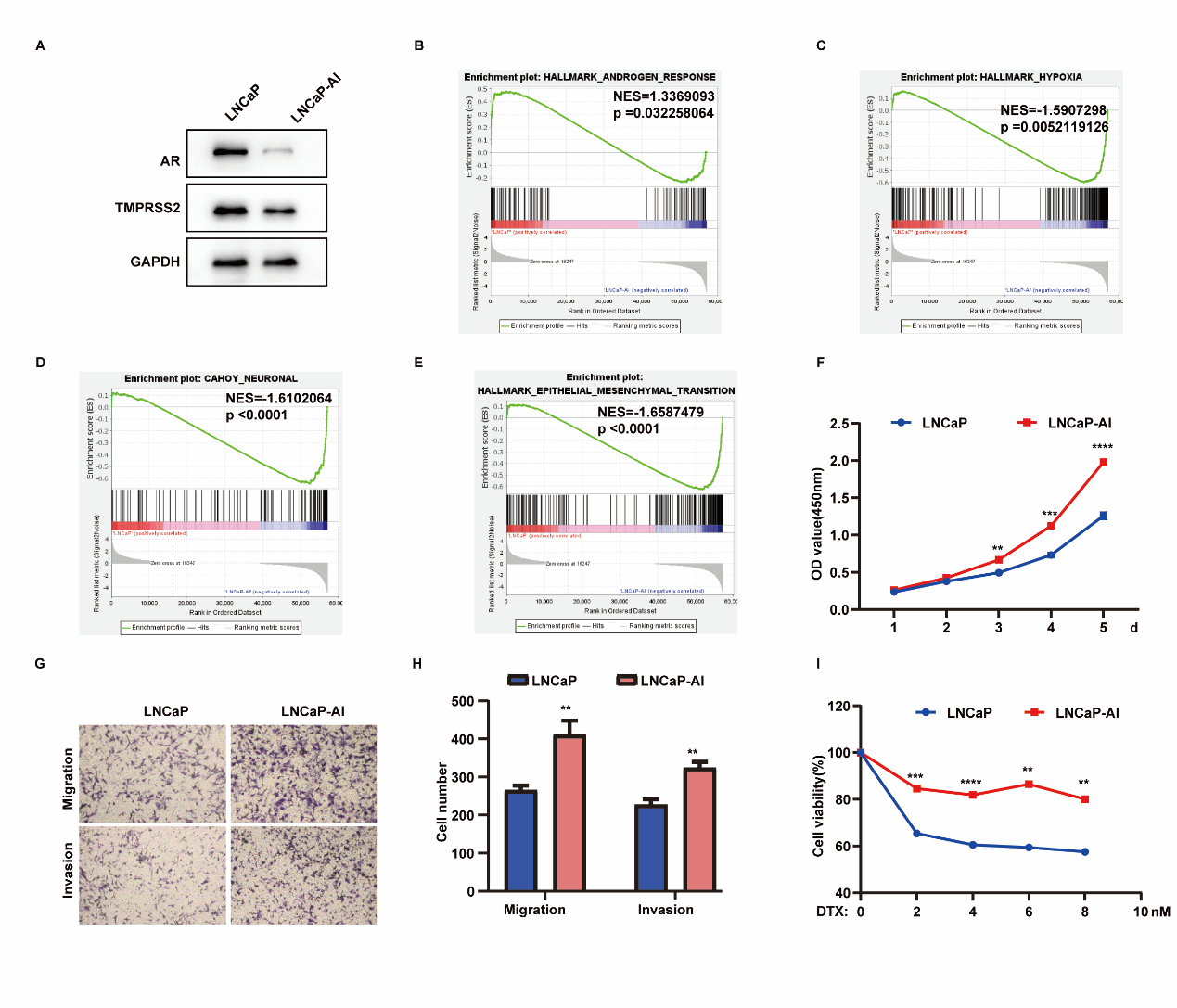


**Supplementary Figure 3. KIF1A expression is upregulated in NE transdifferentiated PCa cells**

A. The protein levels of AR and TMPRSS2 in LNCaP or LNCaP-AI cells. B-E. GSEA of the AR response/ hypoxia /neuronal/ EMT gene signatures in LNCaP and LNCaP-AI cells. F. Cell viability assessed by CCK-8 assay of LNCaP or LNCaP-AI cell. G. Cell migration and invasion evaluated by transwell migration and matrigel invasion assays. H. Quantitative results of migration and invasion assays from three independent experiments. I. Cell viability measured in the indicated cell lines by CCK-8 assay. LNCaP and LNCaP-AI cells were treated with titrated doses of DTX for 3 days. All results were presented as the mean ± SD. **p< 0.01, ***p< 0.001, ****p<0.0001, based on Student’s t test. DTX, docetaxel.


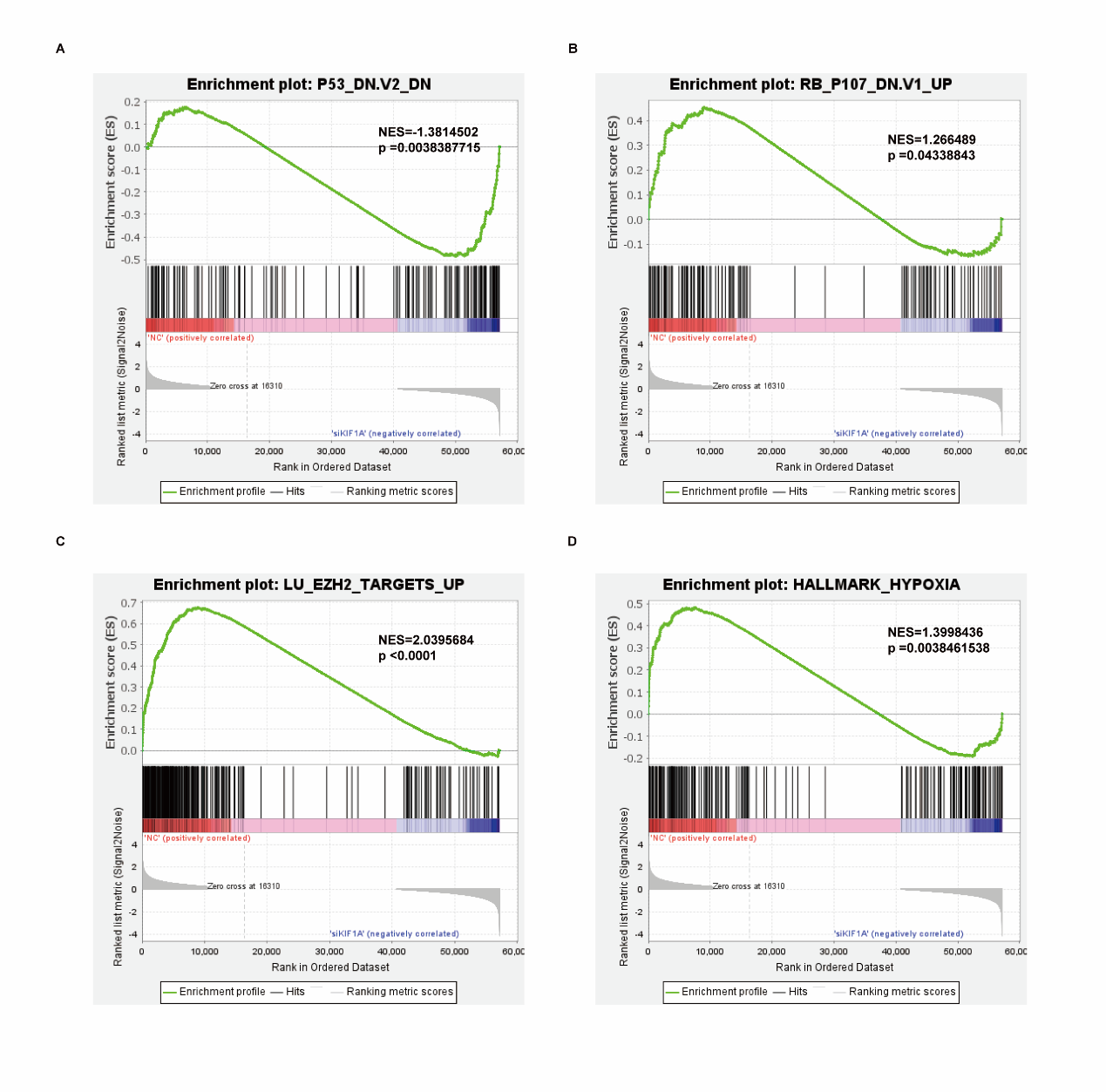


**Supplementary Figure 4. GSEA analysis of differentially expressed genes induced by siKIF1A**

A-E. Enrichment of NE-related gene signatures analyzed by GSEA. LNCaP-AI cells were transfected with indicated siRNAs for 3 days. Total RNA was collected and used to perform microarray-based human gene expression profiling. GSEA was carried out to examine the enrichment of the P53 DN/ RB DN/ EZH2 target/ hypoxia gene sets. DN, knockdown.


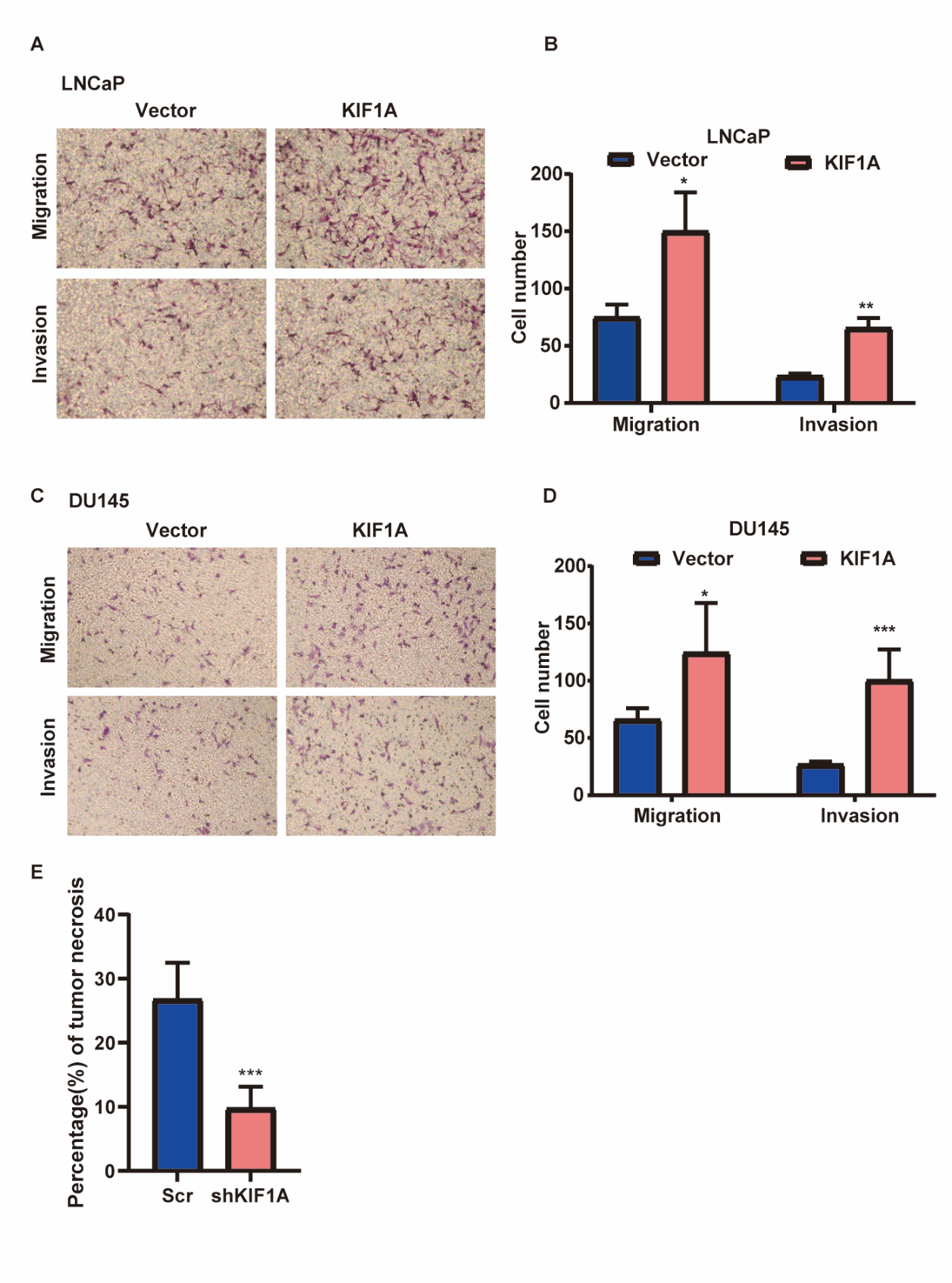


**Supplementary Figure 5. KIF1A promotes aggressive growth of of PCa cell in vitro and in vivo.**

A. Representative images of transwell migration and matrigel invasion assays in indicated LNCaP cells. B. Quantitative results of the transwell migration and matrigel invasion assays from triplicate experiments. C. Representative images of transwell migration and matrigel invasion assays in indicated DU145 cells. D. Quantification of the transwell migration and matrigel invasion assays in indicated DU145 cells from triplicate experiments. E. The percentage of tumor necrosis in LNCaP-AI xenograft tumors with stable expression of Scr/shKIF1A. Sections were stained with H&E staining to identify necrotic tumor. Whole-field images of xenograft tumors were obtained with SCN 400 Leica scanner at 40× magnification. Image J (version 1.8.0; National Institutes of Health) was used to measure the area of tumor and necrotic areas. All results were presented as the mean ± SD of three independent experiments. *p< 0.05, **p< 0.01, ***p< 0.001, based on Student’s t test.


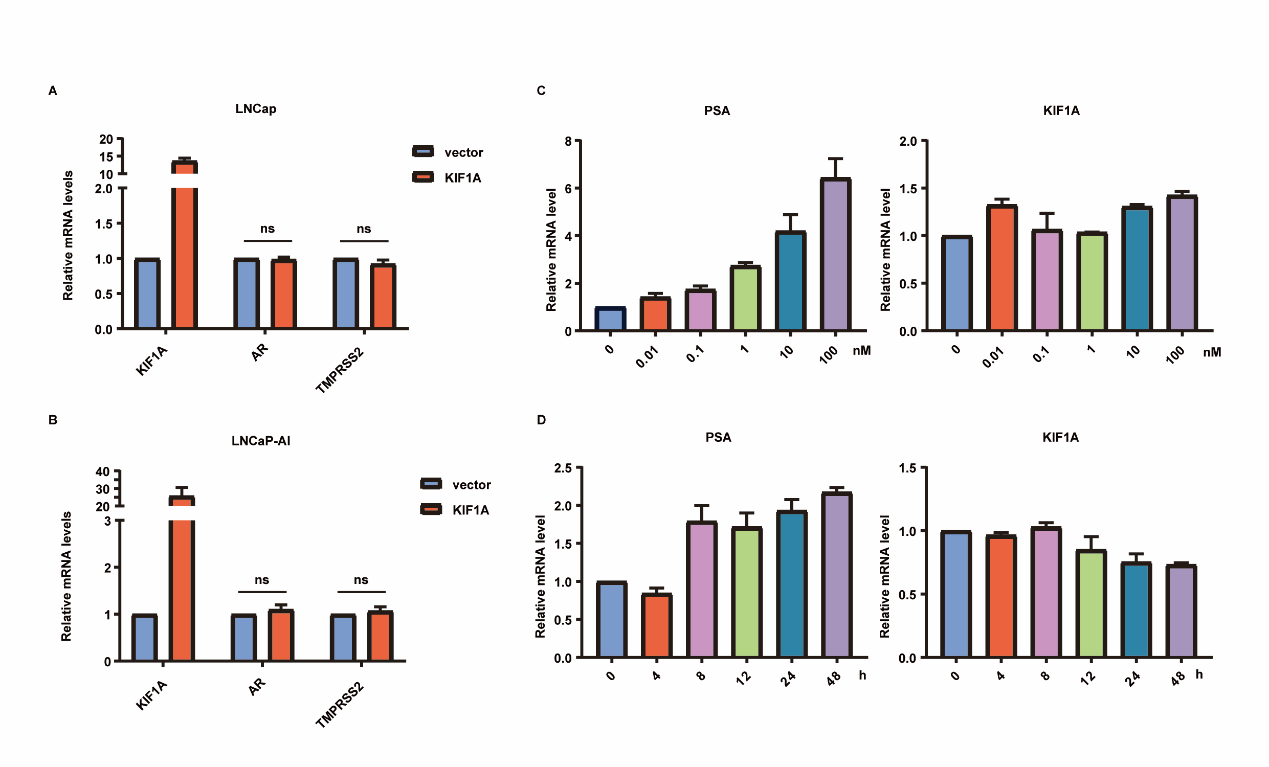


**Supplementary Figure 6**. **No regulation cascade between AR signaling and KIF1A expression.**

A-B. The mRNA levels of AR and TMPRSS2 in LNCaP/LNCaP-AI cells with KIF1A overexpression measured by qPCR assays. C-D. The mRNA levels of KIF1A in DHT-stimulated LNCaP cells. LNCaP cells were cultured in charcoal stripped serum for 3 days before challenged with 0–100 nM DHT for 24 h or 1 nM DHT for 0–48 h. All results were presented as the mean ± SD of three independent experiments. ns indicates no statistical significance, based on Student’s t test.


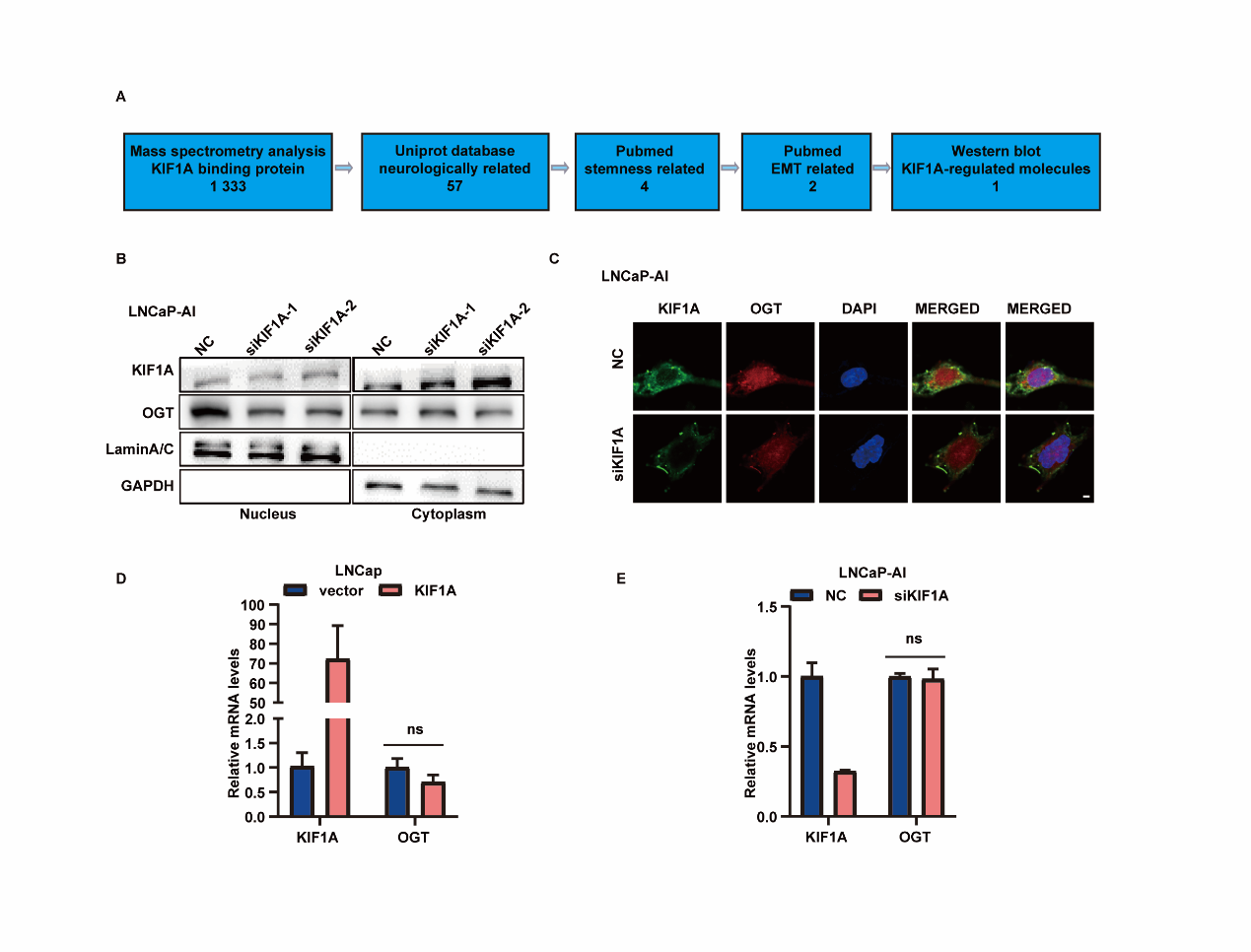


**Supplementary Figure 7. Identification of OGT as conjugated protein of KIF1A**

A. Schematic illustration of molecular screening downstream of KIF1A. B. Nuclear/cytoplasmic expression of OGT and KIF1A in LNCaP-AI cells with KIF1A knockdown. C. KIF1A (green) and OGT (red) expression detected by immunofluorescence in LNCaP-AI cells with KIF1A knockdown. siKIF1A-2 were utilized as siKIF1A. Cells were imaged by confocal microscopy. Scale bar = 5μm. D-E. The mRNA levels of OGT in indicated PCa cells with overexpression/knockdown of KIF1A. All results were presented as the mean ± SD of three independent experiments. ns indicates no statistical significance, based on Student’s t test.


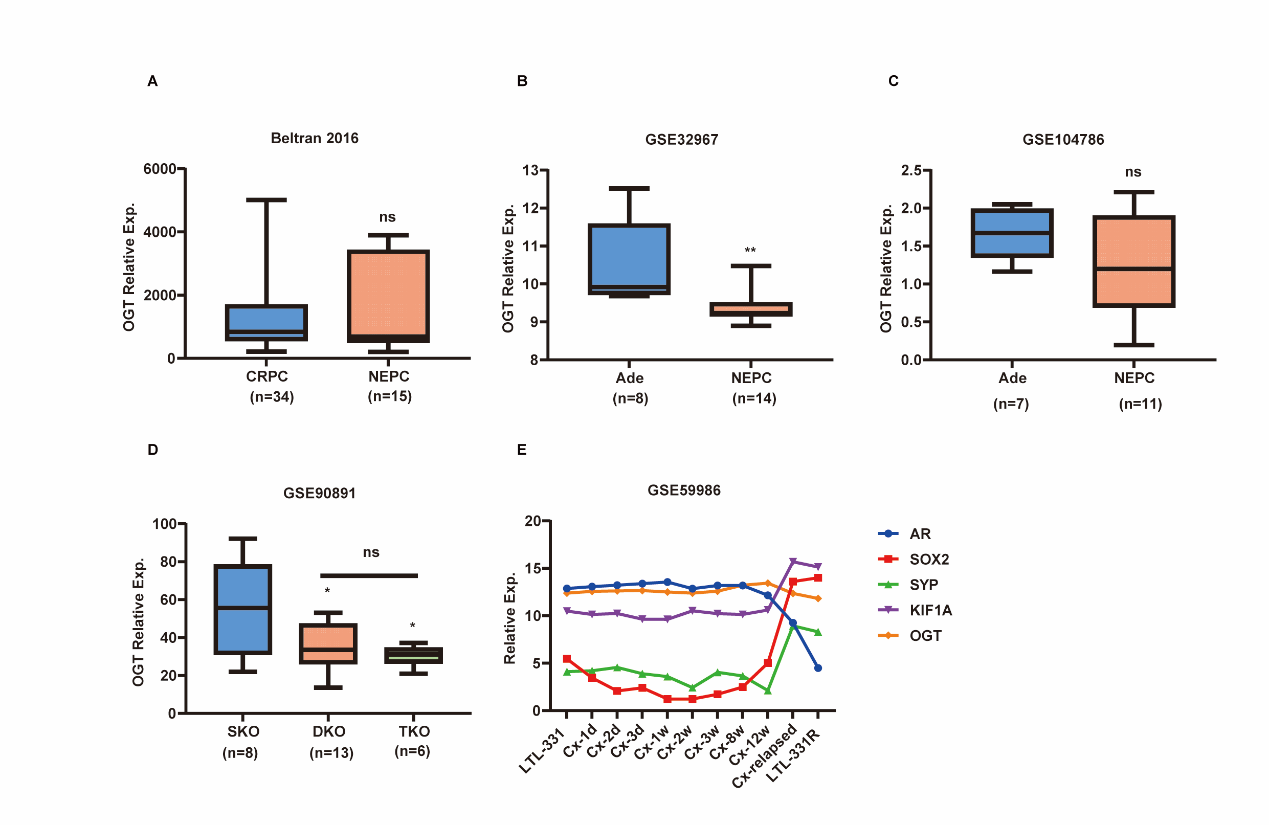


**Supplementary Figure 8. OGT mRNA levels are not statistically different in NE-related groups vs. control in NEPC datasets**

A-C. Expression of KIF1A in NEPC compared with CRPC or Ade. D. Expressions of OGT, KIF1A, SOX2, AR, and SYP during the progression of Ade (LTL331) to t-NEPC (LTL331R-NE) after castration in the PDX LTL331/331R model. E. Expressions of OGT in different GEMM in GSE90891 dataset. All results were presented as the mean ± SD. * p< 0.05, ** p< 0.01, *** p< 0.001. **** p<0.0001. Ade, adenocarcinoma; NEPC, neuroendocrine prostate cancer; SKO, single knockout; DKO, double knockout; TKO, triple knockout


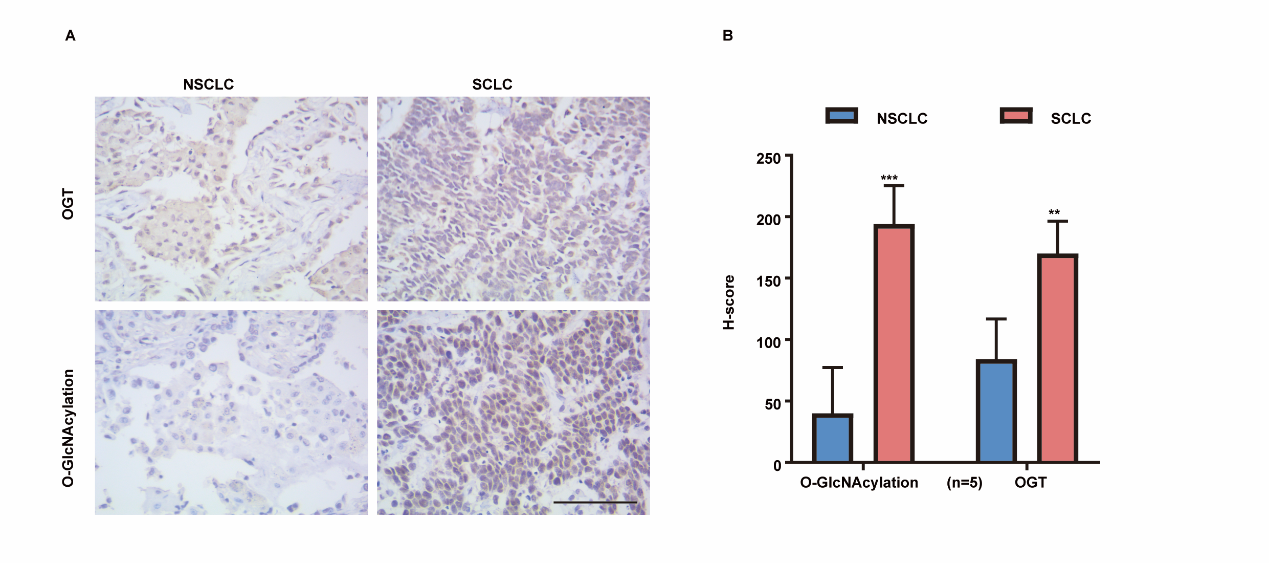


**Supplementary Figure 9. Immunohistochemistry of O-GlcNAcylation and OGT in NSCLS and SCLC case**

A. Representative images showing immunohistochemistry staining for O-GlcNAcylation and OGT inNSCLC and SCLC case. Scar bar =100μm. B. Quantitative result of the assays. ** p< 0.01, *** p< 0.001, based on Student’s t test. NSCLC, non-small cell lung cancer. SCLC, small cell lung cancer.


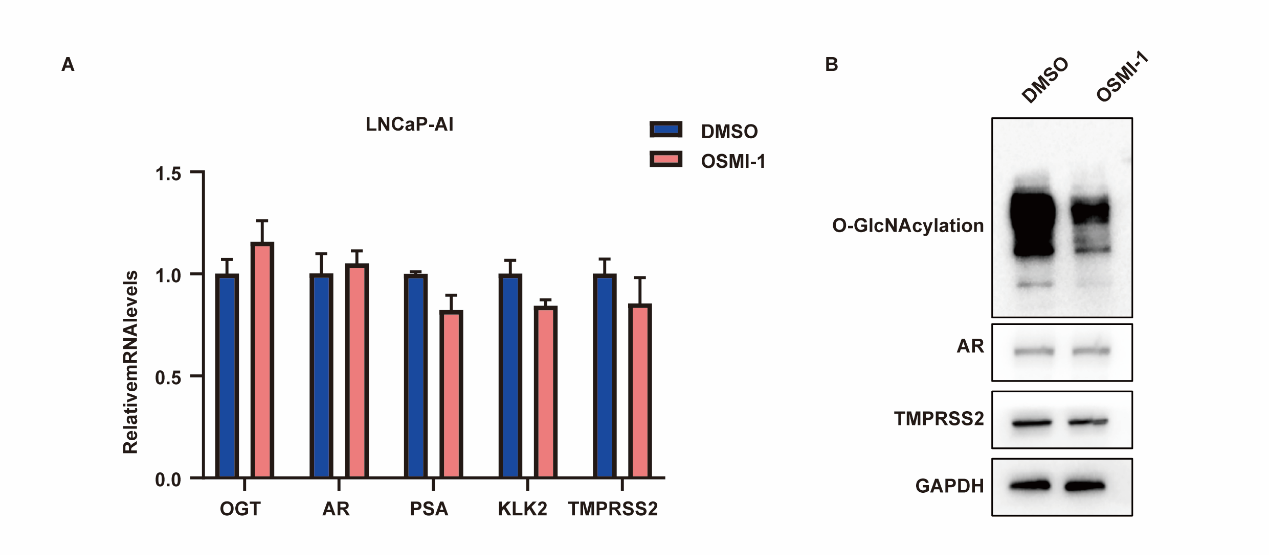


**Supplementary Figure 10. The AR signaling pathway in LNCaP-AI cells is independent of OGT O-glycosyltransferase activity.**

A. The protein levels of O-GlcNAcylation, AR and TMPRSS2 in DMSO/OSMI-1-stimulated LNCaP-AI cells. LNCaP-AI cells were treated with 20μM OSMI-1or DMSO for 72h. B. The mRNA levels of AR signaling pathway genes in DMSO/OSMI-1-stimulated LNCaP-AI cells. LNCaP-AI cells were treated with 20μM OSMI-1or DMSO for 72h.All results were presented as the mean ± SD. of three independent experiments.
