# Supplementary material for: KIF1A promotes neuroendocrine differentiation in prostate cancer by regulating the OGT-mediated O-GlcNAcylation
Source: Cell Death Dis. 2024 Nov 6;15(11):796. doi: 10.1038/s41419-024-07142-2 (PMC11542072; doi:10.1038/s41419-024-07142-2)

Figure1

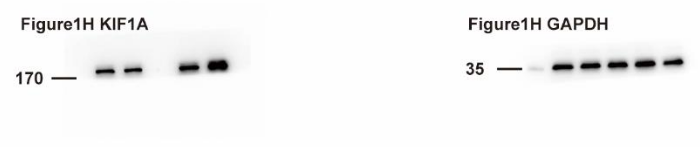

Figure2

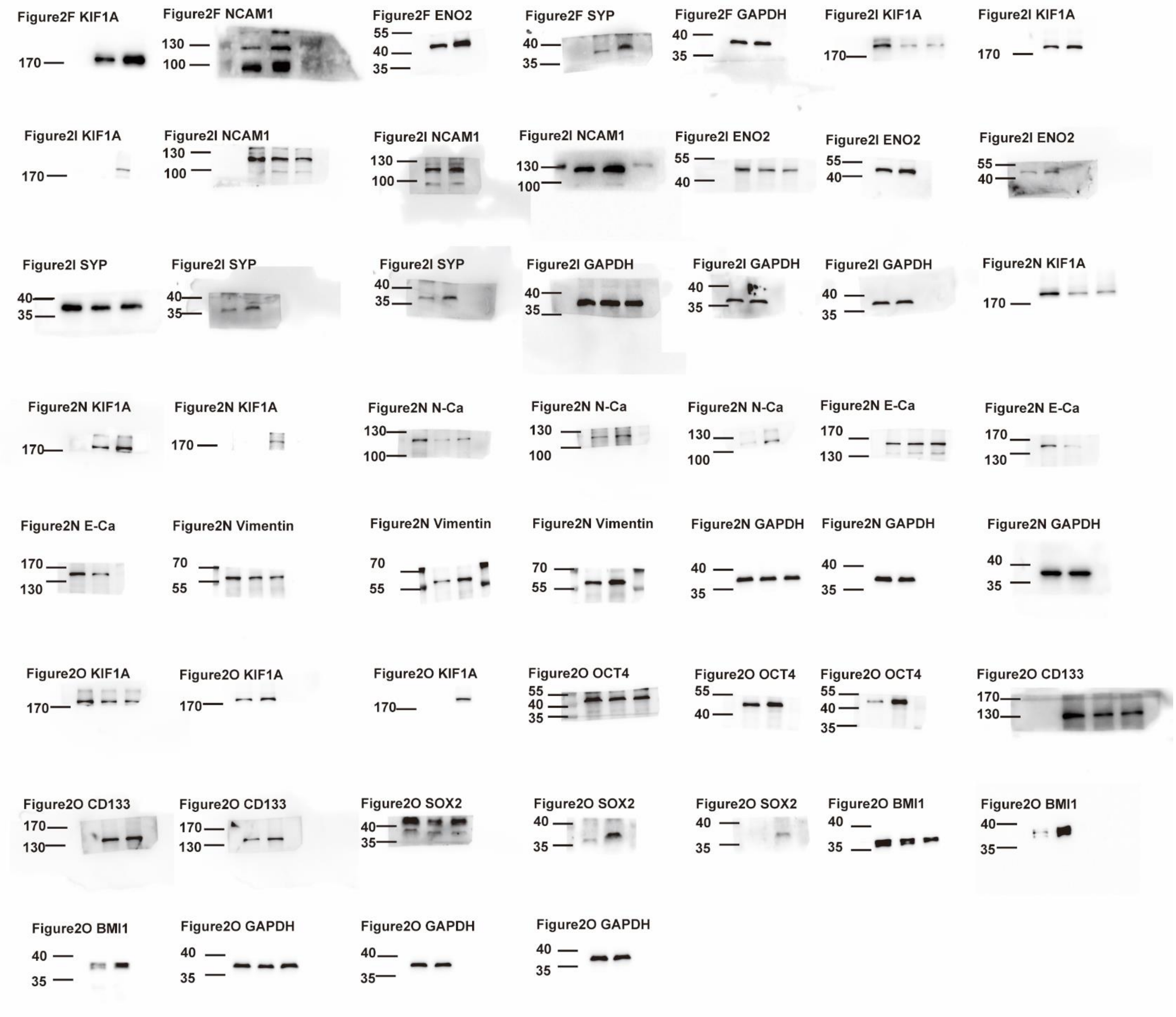

Figure3

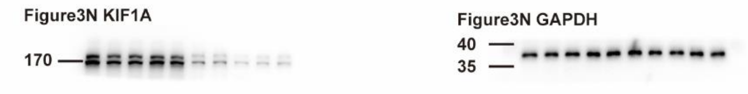

Figure 4

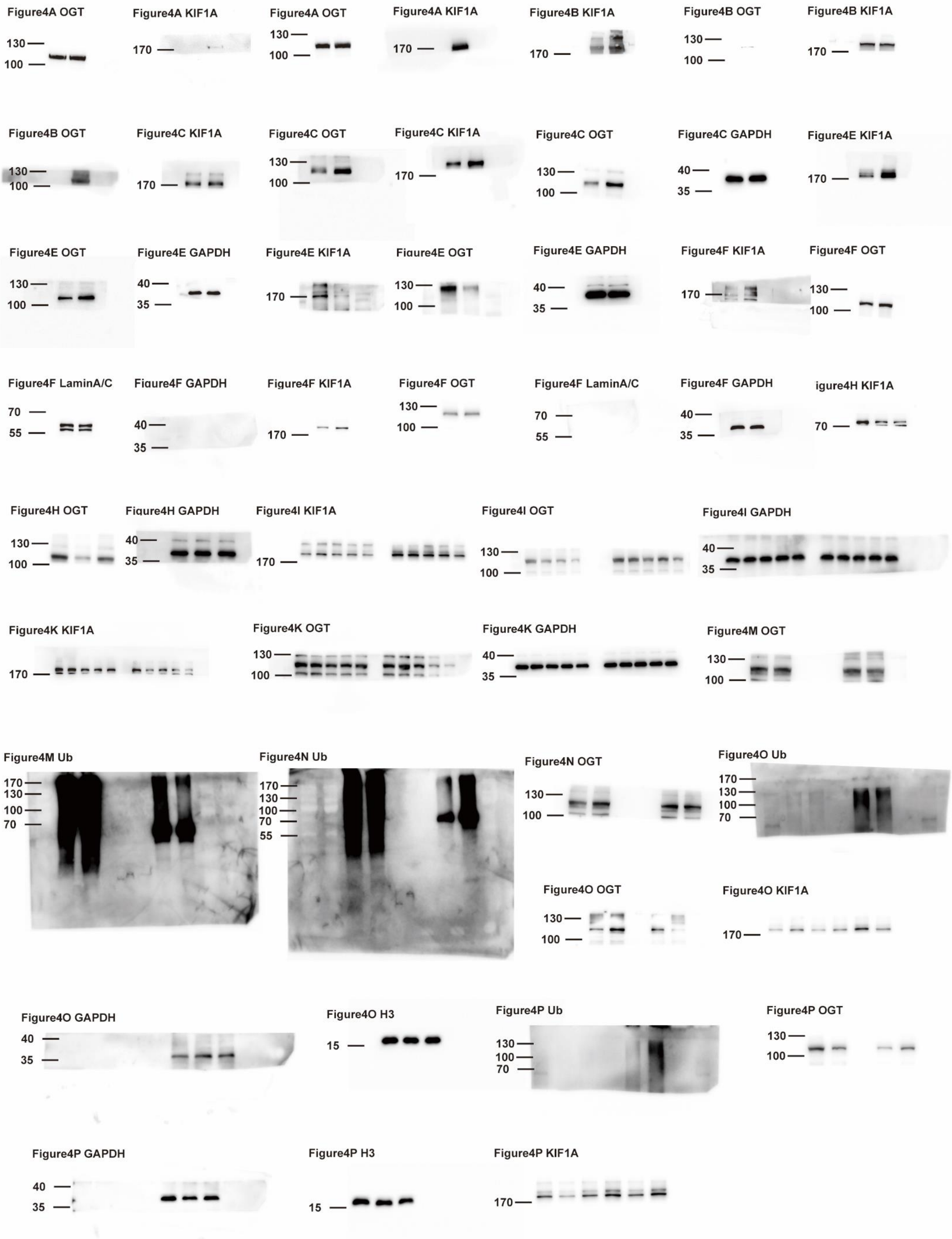

Figure4A OGT
130
100
Figure4A KIF1A
170
Figure4A OGT
130
100
Figure4A KIF1A
170
Figure4B KIF1A
170
Figure4B OGT
130
100
Figure4B KIF1A
170
Figure4B OGT
130
100
Figure4C KIF1A
170
Figure4C OGT
130
100
Figure4C KIF1A
170
Figure4C OGT
130
100
Figure4C GAPDH
40
35
Figure4E KIF1A
170
Figure4E OGT
130
100
Figure4E GAPDH
40
35
Figure4E KIF1A
170
Figure4E OGT
130
100
Figure4E GAPDH
40
35
Figure4F KIF1A
170
Figure4F OGT
130
100
Figure4F LaminA/C
70
55
Figure4F GAPDH
40
35
Figure4F KIF1A
170
Figure4F OGT
130
100
Figure4F LaminA/C
70
55
Figure4F GAPDH
40
35
igure4H KIF1A
70
Figure4H OGT
130
100
Figure4H GAPDH
40
35
Figure4I KIF1A
170
Figure4I OGT
130
100
Figure4I GAPDH
40
35
Figure4K KIF1A
170
Figure4K OGT
130
100
Figure4K GAPDH
40
35
Figure4M OGT
130
100
Figure4M Ub
170
130
100
70
Figure4N Ub
170
130
100
70
55
Figure4N OGT
130
100
Figure4O Ub
170
130
100
70
Figure4O OGT
130
100
Figure4O KIF1A
170
Figure4O GAPDH
40
35
Figure4O H3
15
Figure4P Ub
130
100
70
Figure4P OGT
130
100
Figure4P GAPDH
40
35
Figure4P H3
15
Figure4P KIF1A
170

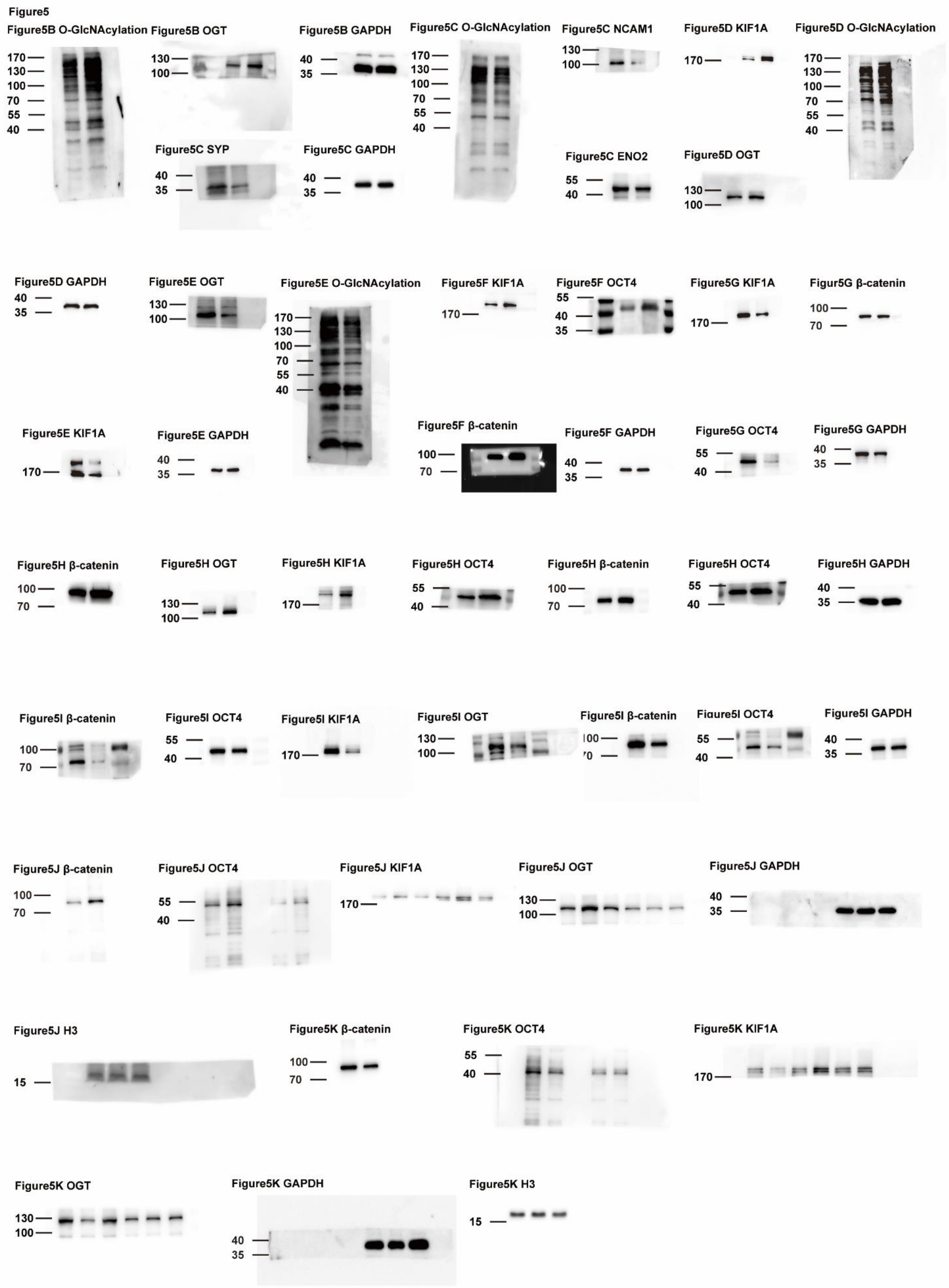
Figure5
Figure5B O-GlcNAcylation
170
130
100
70
55
40
Figure5B OGT
130
100
Figure5B GAPDH
40
35
Figure5C O-GlcNAcylation
170
130
100
70
55
40
Figure5C NCAM1
130
100
Figure5D KIF1A
170
Figure5D O-GlcNAcylation
170
130
100
70
55
40
Figure5C SYP
40
35
Figure5C GAPDH
40
35
Figure5C ENO2
55
40
Figure5D OGT
130
100
Figure5D GAPDH
40
35
Figure5E OGT
130
100
Figure5E O-GlcNAcylation
170
130
100
70
55
40
Figure5F KIF1A
170
Figure5F OCT4
55
40
35
Figure5G KIF1A
170
Figur5G β-catenin
100
70
Figure5E KIF1A
170
Figure5E GAPDH
40
35
Figure5F β-catenin
100
70
Figure5F GAPDH
40
35
Figure5G OCT4
55
40
Figure5G GAPDH
40
35
Figure5H β-catenin
100
70
Figure5H OGT
130
100
Figure5H KIF1A
170
Figure5H OCT4
55
40
Figure5H β-catenin
100
70
Figure5H OCT4
55
40
Figure5H GAPDH
40
35
Figure5I β-catenin
100
70
Figure5I OCT4
55
40
Figure5I KIF1A
170
Figure5I OGT
130
100
Figure5I β-catenin
100
70
Figure5I OCT4
55
40
Figure5I GAPDH
40
35
Figure5J β-catenin
100
70
Figure5J OCT4
55
40
Figure5J KIF1A
170
Figure5J OGT
130
100
Figure5J GAPDH
40
35
Figure5J H3
15
Figure5K β-catenin
100
70
Figure5K OCT4
55
40
Figure5K KIF1A
170
Figure5K OGT
130
100
Figure5K GAPDH
40
35
Figure5K H3
15

Figure6

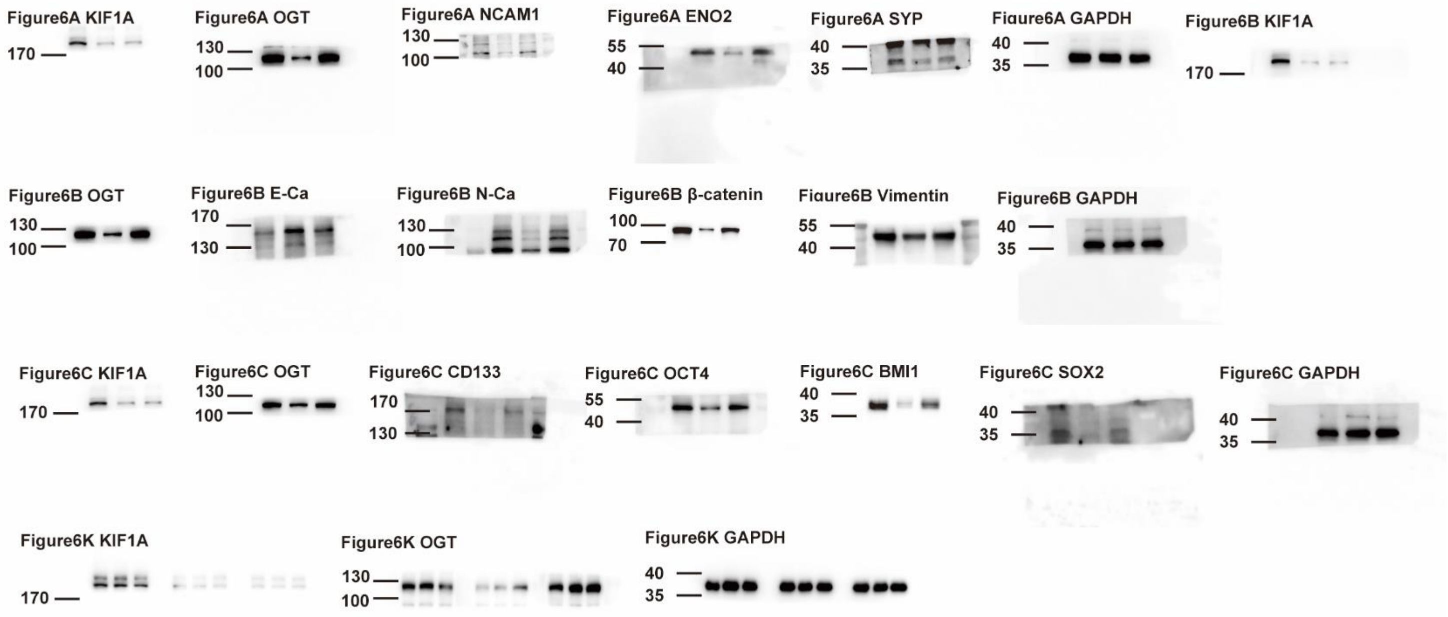

Supplementary Figure3

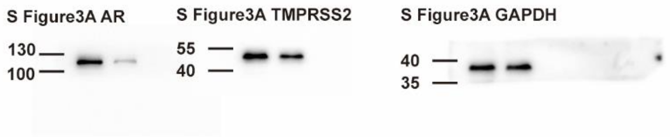

Supplementary Figure7

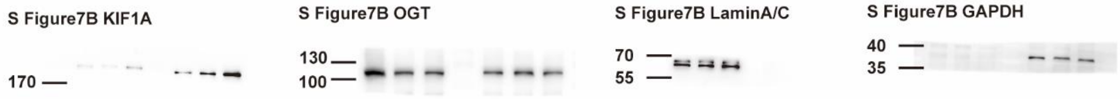

Supplementary Figure10

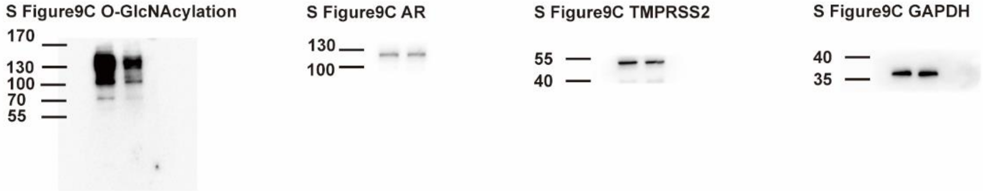

Supplement: Supplementary file 4 — Orignal Western blots [file 41419_2024_7142_MOESM4_ESM.pdf]
